# Genome-Wide and Experimental Resolution of Relative Translation Elongation Speed at Individual Gene Level in Human Cells

**DOI:** 10.1371/journal.pgen.1005901

**Published:** 2016-02-29

**Authors:** Xinlei Lian, Jiahui Guo, Wei Gu, Yizhi Cui, Jiayong Zhong, Jingjie Jin, Qing-Yu He, Tong Wang, Gong Zhang

**Affiliations:** Institute of Life and Health Engineering, Key Laboratory of Functional Protein Research of Guangdong Higher Education Institutes, Jinan University, Guangzhou, China; University of Maryland Medical School, UNITED STATES

## Abstract

In the process of translation, ribosomes first assemble on mRNAs (translation initiation) and then translate along the mRNA (elongation) to synthesize proteins. Elongation pausing is deemed highly relevant to co-translational folding of nascent peptides and the functionality of protein products, which positioned the evaluation of elongation speed as one of the central questions in the field of translational control. By integrating three types of RNA-seq methods, we experimentally and computationally resolved elongation speed, with our proposed elongation velocity index (EVI), a relative measure at individual gene level and under physiological condition in human cells. We successfully distinguished slow-translating genes from the background translatome. We demonstrated that low-EVI genes encoded more stable proteins. We further identified cell-specific slow-translating codons, which might serve as a causal factor of elongation deceleration. As an example for the biological relevance, we showed that the relatively slow-translating genes tended to be associated with the maintenance of malignant phenotypes per pathway analyses. In conclusion, EVI opens a new view to understand why human cells tend to avoid simultaneously speeding up translation initiation and decelerating elongation, and the possible cancer relevance of translating low-EVI genes to gain better protein quality.

## Author Summary

In protein synthesis, ribosome assembles to mRNA to initiate translation, followed by the process of elongation to read the codons along the mRNA molecule for polypeptide chain production. It is known that slowing down the elongation speed at certain regions of mRNA is critical for the correct folding of numerous proteins—the so-called “pause-to-fold”. However, it has been an open question to evaluate elongation speed under cellular physiological conditions in genome-wide scale. Here, we used three types of next-generation sequencing approaches to experimentally and computationally address this question. With a new relative measure of elongation velocity index (EVI), we successfully distinguished slow-translating genes. Their protein products are more stable than the background genes. We found that different cell types tended to have distinct slow-translating codons, which might be relevant to the cell/tissue specific tRNA composition. Such elongation deceleration is potentially disease-relevant: cancer cells tend to slow down numerous cancer-favorable genes, and *vice versa*. Furthermore, we justified that translation initiation and elongation are evolutionarily synergistic as no gene with both high initiation efficiency and low elongation speed was observed: that would cause a traffic jam of ribosomes that should be maximally avoided per evolution.

## Introduction

Protein synthesis is a collective outcome of different mechanisms of translation control, including: i) translation initiation, namely the assembly process of methionyl-tRNA loaded and elongation-competent 80S ribosomes at mRNA start (AUG) codons (reviewed in ref. [1–4]), which determines the fraction of mRNA molecules that can be translated [5, 6]; ii) translation elongation, which determines the translating speed of ribosome(s) post-initiation on a single mRNA molecule (reviewed in ref. [7, 8]); and iii) termination that allows the reuse of ribosomes.

Indeed, elongation speed with its biological relevance is one of the most well-known challenges in understanding translational control, especially regarding cells under physiological conditions. If not considering pre-mature termination (ribosome drop-off) and frameshifting, elongation speed is in general independent from the abundance of protein products, at least in mouse embryonic stem cells [9]. This is comparable with numerous reports showing that initiation is the rate-limiting step of translation in eukaryotic cells [5, 10, 11] (reviewed in ref. [2, 7]), which we have computationally modeled the length-dependent initiation by considering the circulation time and its impact on maintaining a functional proteome [12]. Regarding elongation, we previously experimentally validated that the major determinants of elongation speed should include codon selection and cognate tRNA abundance [13]. Interestingly, we and others have reported that the abundance of tRNAs decoding different codons may vary more than one order of magnitude, thus the elongation speed should be non-uniform even on a single mRNA molecule [13–17].

The biological significance of elongation speed control has been emphasized to understand human disease such as cancer, hemophilia and hyperactivity disorder (reviewed in ref. [18]). Kimchi-Sarfaty *et al* have found that a synonymous mutation of the multi-drug resistance 1 gene (*MDR1*) can cause structural and functional alteration of the gene product that may be relevant to the timing of co-translational folding as hypothesized by the authors [19]. This notion was supported by our previous experimental findings on the translational pausing caused by clusters of slow-translating codons coordinates protein synthesis and co-translational folding that are critical for protein quality [13] (reviewed in ref. [7, 20–22]).

Although several studies from us and others have monitored the elongation speed of single genes [13, 23–25], the genome-wide elongation speed evaluation on eukaryotic cells under physiological conditions is still an open question. Since decades ago, mathematical indices, including codon bias index (CBI), codon adaptation index (CAI), effective number of codons (Nc) and tRNA adaptation index (tAI) have been widely employed to estimate translation elongation efficiency (reviewed in ref. [26]). Most of these indices are calculated using codon usage and tRNA gene copy number of the whole genome, which persist the same in a certain organism. Here, codon usage is a term with strict definition: the occurrence of a certain codon among all codons used in a certain organism [27]. Indeed, the tRNA concentrations for each codon, including the ratio of cognate to near-cognate, is an important determinant of the elongation speed [28, 29]. However, the tRNA concentration correlates poorly to these indices and can vary dramatically in different tissues and cell types, serving as a primary causal factor of tissue-specific translation profiles (reviewed in ref. [20, 26]). With this regard, we previously established an algorithm to use tRNA abundance to predict the elongation speed and translational pausing sites, which was experimentally validated both in single genes and at genome-wide level [13, 14, 30]. Unfortunately, absolute tRNA abundance information at anticodon resolution is currently unavailable for higher eukaryotes due to the technological hindrance to resolve the high complexity and homology of tRNA molecules (reviewed in [20]). Other than tRNA concentration, it has been investigated whether the mRNA secondary structure, steric effect of tRNAs and amino acid charge are major determinants of elongation velocity. In the view of computational biology, the mRNA secondary structure may affect the elongation speed [16], supported by the pseudoknot-directed translational frameshifting as an example [31]. In contrast, other evidence acquired by the pulse-chase labeling and single ribosome monitoring suggested that stable mRNA secondary structures do not cause any translation delay [24, 25]. Despite of diversified structures of tRNAs, it was proposed that all of the aminoacyl-tRNAs are selected uniformly on the ribosome, suggesting that steric effects have minimum influence on the elongation speed [32]. Interestingly, repeats of positively charged amino acids have been found to cause translational pausing [16, 33, 34]; furthermore, singlets of such amino acids do not necessarily cause translational deceleration [35]. In addition, other specific sequences, such as anti-Shine-Dalgarno sequence and stalling nascent peptides, have been found to pause the ribosomes regarding some specific genes [36, 37].

Due to such complex scenarios, to directly assess the translation elongation speed in a high-throughput manner is necessary. Ribosome profiling provides a detailed snapshot of ribosome occupancy [38], making it feasible to study the ribosome density profile of translation ([9] and reviewed in ref. [8]). Ingolia *et al* monitored the progression of the average profiles of ribosome footprints (RFPs) and revealed an average translation elongation speed of 5.6 codons/sec in mouse embryonic stem cells; however, this measurement of ribosome elongation has a 60-s delay caused by the harringtonine treatment [9].

**Fig 1:**
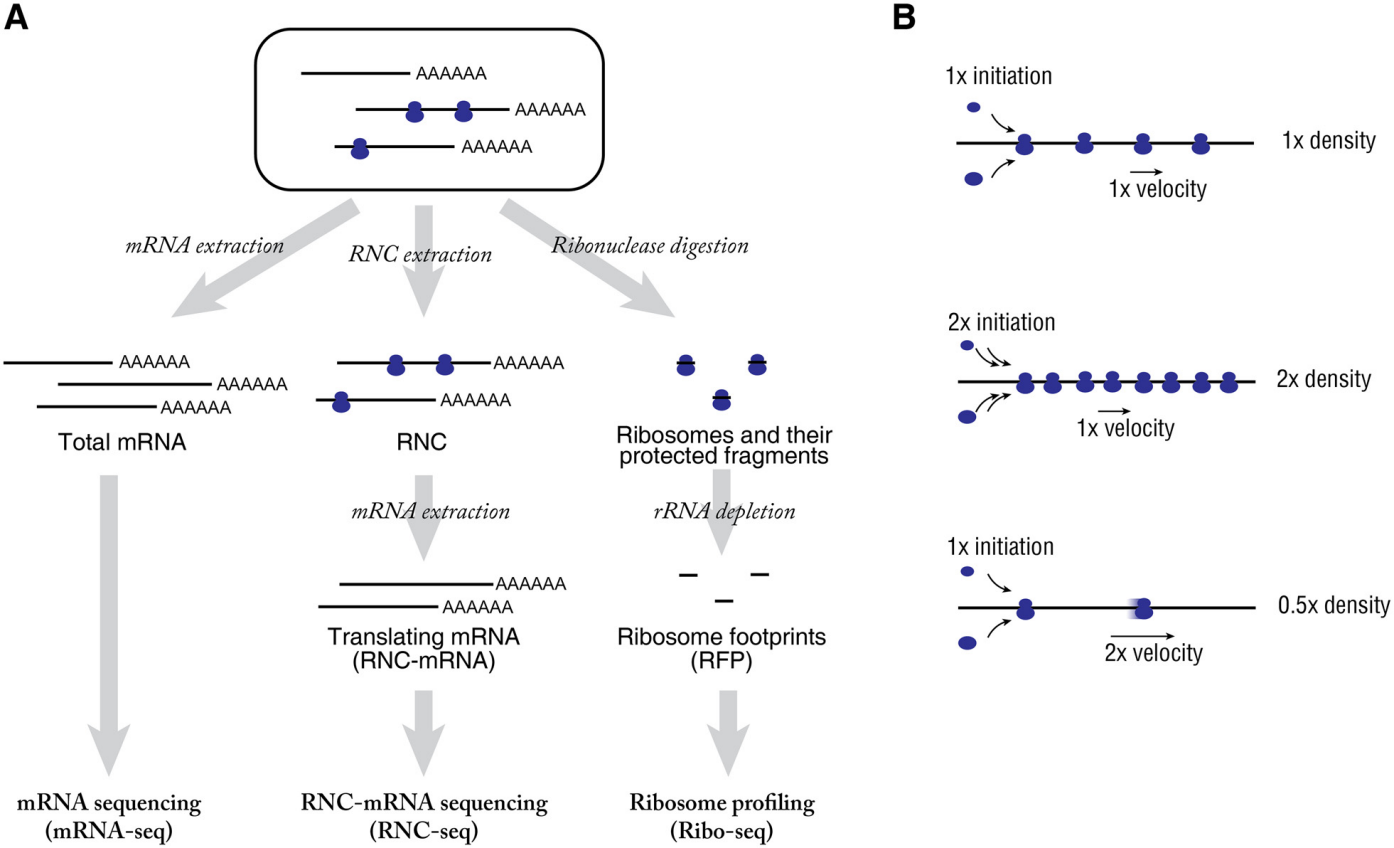
Measurement of TR and EVI. (A) Schematic workflow of mRNA-seq, RNC-seq and Ribo-seq of the same batch of cultured cells. (B) Contribution of translation initiation efficiency and elongation velocity to the ribosome density.

We previously reported a strategy to combine the full length sequencing on ribosome nascent-chain complex (RNC) bound mRNA (RNC-mRNA) and total mRNA for the global translation initiation investigation [6, 39]; we showed that the translation ratio (TR, abundance ratio of RNC-mRNA/mRNA for a certain gene) can properly reflect cellular phenotypes. In this study, we integrated three types of current RNA-seq strategies, including mRNA sequencing (mRNA-seq), full-length RNC-mRNA sequencing (RNC-seq) and ribosome profiling (Ribo-seq) (Fig 1A). As an outcome, we resolved global elongation speed by an Elongation Velocity Index (EVI) at individual gene level in human normal and cancer cells under physiological conditions. This allowed us to distinguish slow-translating genes and codons in different human cell lines, respectively. Furthermore, our results favored the hypothesis on the cancer relevance of co-translational folding by providing the experimental and computational evidence on a genome-wide scale.

## Results

### Estimation of relative translation velocity by the Elongation Velocity Index

Using reads per kilo base per million (rpkM) as unit, the abundance of mRNA (M), RNC-mRNA (C) and RFP (F) are length-independent. Therefore, the RNC-mRNA ribosome density (Density), which is defined here as F/C, and TR that is defined as C/M [6] can be compared between different genes. Here, RNC-mRNA ribosome density is not a constant, it varies drastically on genome-wide scale either in a single cell type (S1A Fig) or across different cell types (S1B Fig). As the translation initiation is the rate-limiting factor of the entire translation process in eukaryotes [5], TR is a relative measure of the translation initiation efficiency in eukaryotes [6, 12, 39]. Here, we defined the translation initiation efficiency as the ratio of the amount of mRNAs that are being translated to the amount of transcribed mRNAs of a certain gene [6]. RNC-mRNA ribosome density is proportional by the ratio of translation initiation efficiency and elongation speed (Fig 1B). Here, we define the “Elongation Velocity Index” (EVI) as a relative measure of elongation speed to make

$$Density=\frac{F}{C}=\frac{TR}{EVI}\;.$$ (1)

Thus:

$$EVI=\frac{TR\cdot C}{F}=\frac{C^{2}}{M\cdot F}$$ (2)

The TR and EVI values of all of the tested cell lines are provided in S1 Table.

### Genome-wide resolution of translation elongation speed at individual gene level under physiological condition

**Fig 2:**
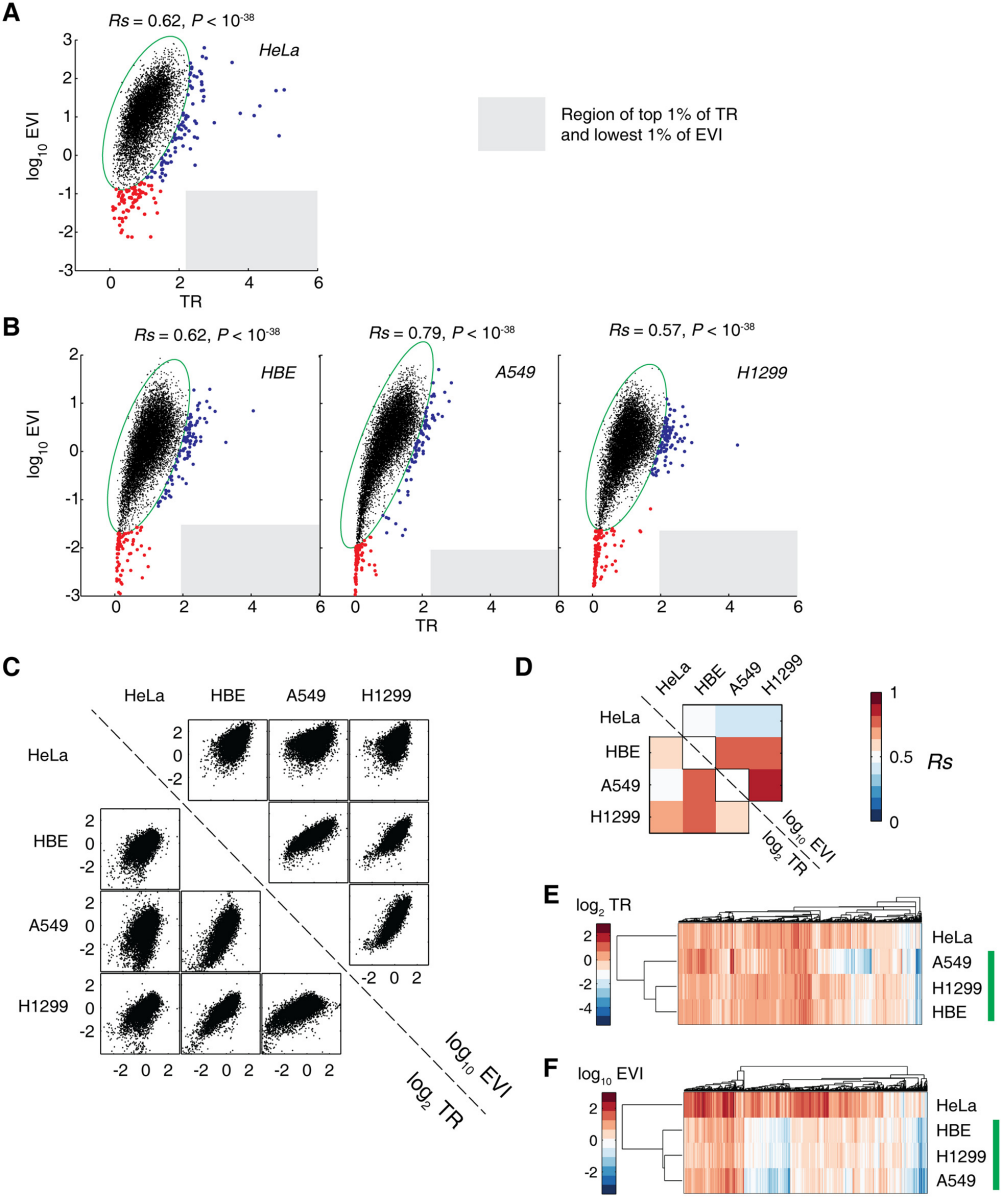
Two mutually exclusive and polarized translation control modes. (A) The TR and EVI of genes in HeLa cells. The green ellipse indicates the 95% confidence ellipse. Genes outside of this ellipse were clustered in two major clusters (red and blue dots) based on their Euclidean distances. The grey region denotes the region with high TR (genes with top 1% TR) and low EVI (genes with lowest 1% EVI), simultaneously. *Rs* = Spearman *R*. (B) The TR and EVI of genes of HBE, A549 and H1299 lung cell lines, respectively. (C) Plot matrix of mutual correlation of the TR (lower half triangle) and EVI (upper half triangle) among the four analyzed cell lines. (D) The heatmap of the Spearman *R* of the panel (C). All of the *P*-values are less than 10^−38^. (E,F) Cluster analysis of the TR (E) and EVI (F) of the four cell lines. The lung-derived cell lines are indicated by a green bar.

Due to the possible association of elongation speed and protein degradation, plus the protein turnover rate of HeLa cells has been investigated intensively [40], we used this cell line for the EVI evaluation and verification as an initial step. We detected a total of 10,837 genes in HeLa cells that could be quantified from all of the three types of RNA-seq (Fig 1 and S2 Table). Although TR and EVI emphasize on different stages of translational control, we found they significantly correlated to each other, with the Spearman *R* (*R*s) = 0.62 (*P*<10^−38^; Fig 2A). This suggests that evolutionarily synergistic roles of translational control may exist to prevent certain detrimental effects in translation. To further probe such roles, we computed the 99% Hotelling's T^2^ confidence ellipse and genes outside the confidence ellipse were considered as outliers, which were then subjected to clustering analysis based on the Euclidean distance and Ward’s linkage (Fig 2A). Interestingly, we observed two polarized clusters of outlier genes, namely the low EVI cluster (red dots) and the high TR cluster (blue dots). Considering the gene distribution and empirical cumulative density function (CDF) of TR and EVI, we defined a grey quadrant locating genes with the top 1% of TR and the smallest 1% of EVI, simultaneously (Fig 2A and S2 Fig). No gene was found to distribute in such a grey quadrant for HeLa cells (Fig 2A and S2 Fig) as well as HBE, A549 and H1299 cells (Fig 2B). In addition, similar polarized distribution of genes were confirmatively observed in the human lung cell lines (Fig 2B), which helped to rule out the cell-specific bias. Excluding the RFP reads of upstream ORFs (uORFs) almost led no change to the EVI values (S3 Fig).

The mutual correlation coefficient of the analyzed four cell lines ranges from 0.42 to 0.83 (all *P* < 10^−38^; Fig 2C and 2D) based on TR and EVI, respectively. In comparison, such correlation coefficients calculated based on mRNA and RNC-mRNA ranged from 0.77~0.87, while the RFP of HeLa cells are quite different than the lung-derived cells (*Rs* = 0.45~0.41; all *P <* 10^−38^) (S4 Fig). The hierarchical cluster analysis of the TR and EVI of each gene in the four cell lines showed that the three lung-derived cells are clustered together and deviate from HeLa cells (Fig 2E and 2F), suggesting a tissue-specific pattern both on translation initiation efficiency and elongation speed. In addition, we found 49 overlapping low-EVI genes and 4 overlapping high-TR genes among the three lung-derived cell lines (S4A and S4B Table). In contrast, we observed 4 overlapping low-EVI and no overlapping high-TR genes among all of the four analyzed cell lines (S4C Table). This indicates that the low-EVI genes and high-TR genes are tissue-specific, which corresponds to our previous findings that high-TR genes reflect the specific cellular phenotypes and organ origins [6]. Gene ontology (GO) enrichment analyses showed that the low-EVI genes in four cell lines shared some universal and housekeeping biological processes, e.g. cellular component organization (S5 Fig). At the same time, the GO enrichments also exhibited remarkable tissue specificity (S5 Fig).

Notably, D = TR/EVI spans a wide distribution of 4~5 log units in each cell line (S1A Fig). This range is similar to the span of EVI distribution (Fig 2A and 2B). The correlation of D in HeLa and lung cells are at <0.4 level (S1B Fig), suggesting a high variability of RNC-mRNA ribosome densities across the cells. In comparison, higher correlation of densities were detected among the lung-derived cell lines (S1B Fig), which echoed the tissue specificity of translation elongation. Thus, experimental measurement is necessary to test the EVI and TR correlations.

We then calculated CAI [27], CBI [41] and Nc [42, 43] in all of the analyzed cell lines, respectively. We found that EVI has only minor correlations with CAI (|*Rs*| < 0.13; all *P*<10^−5^; S6 Fig) and CBI (|*Rs*| < 0.11; all *P*<10^−8^; S7 Fig), respectively. Interestingly, EVI has significant negative correlation with Nc (*Rs* = -0.15~-0.46; all *Ps* < 10^−38^; S8 Fig).

### Elongation deceleration and protein stability

We have previously validated the phenotypic relevance of high TR [6]; therefore, we next focused on examining whether the slow-translating genes have translational pausing sites and whether they are more stable. Here, we adopted the definition that the highest RFP peaks that higher than 10-fold of average count are translational pausing sites [9].

**Fig 3:**
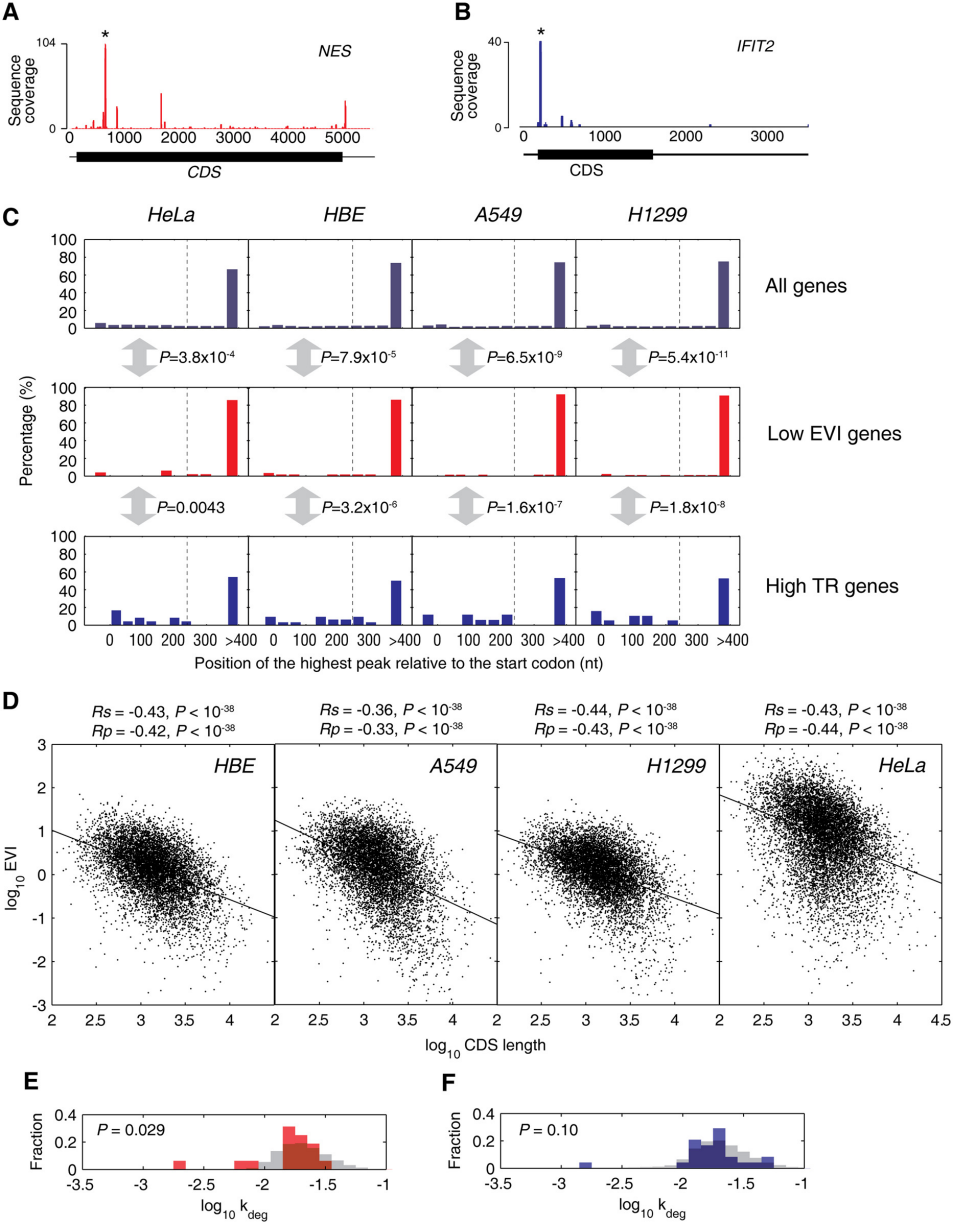
The relevance of low EVI genes to transient translational pausing and protein folding. (A, B) The RFP coverage of a representative gene with low EVI (A) and high TR (B) in HeLa cells, respectively, are shown with color bars. The highest RFP peaks are indicated by stars. The full length mRNAs are shown in thin lines, while their CDS regions are marked with thick lines. For more examples in all four analyzed cell lines, please refer to S6 Fig. (C) The percentage distribution of the highest RFP peak of each gene relative to the start AUG codon. The highest RFP peaks more than 400 nucleotides are counted in the bin of 400. The *P*-values of Kolmogorov-Smirnov test between the low EVI genes and high TR genes as well as all genes in each cell line are indicated. The grey dashed lines mark the 240nt position. (D) Correlation between CDS length and EVI in the four cell lines. (E, F) The distribution of genes in HeLa cells according to their protein degradation rate constant (*k*_deg_). Fractions of the genes with low EVI (E) and high TR (F) were indicated in red and blue bars, respectively. For comparison, the fraction distributions of total genes are shown in grey bars as background.

We found that the RFP distribution of low-EVI genes in HeLa cells, such as *NES*, showed significant translational pausing sites, and their highest RFP peaks predominantly occur more than 240nt after the start codons (Fig 3A). It is known that the pausing sites after the 240nt threshold predominantly coordinate protein synthesis and co-translational folding [13, 14, 44]. In contrast, some high TR genes in HeLa cells, such as *IFIT2*, has the highest RFP peaks located next to the respective start codons, indicating its high efficiency of translation initiation and fewer translational pausing sites (Fig 3B). More examples can be found in the S9 Fig. We statistically confirmed these phenomena observed on the randomly chosen genes (Fig 3C). Approximately half of the high TR genes have their highest RFP peaks prior to 240nt away from the start codons, significantly different from the low EVI genes (*P* = 0.0043~1.8×10^−8^, Fig 3C). Notably, all of the highest RFP peaks in either the low EVI genes or the high TR genes in the four analyzed cell lines have >10-fold more coverage than the average, thus can be considered as true translational pausing sites but not the sequencing bias as explained by Ingolia *et al* [9].

In addition, we found that EVI negatively correlated to CDS length for the genes of the four cell lines, respectively, suggesting that longer genes are generally translated slower in these human cells (Fig 3D). This coincides with our previous findings in bacteria that longer proteins possess more translational pausing sites to overcome the folding complexity of multiple structural domains [5, 13, 45]. Thus, these results independently favored the validity of using EVI to resolve gene elongation speeds.

We next acquired the protein degradation rate constant data of HeLa cells from the report by Cambridge *et al* [40].We found that the proteins encoded by the low EVI outlier genes had 1.3-fold less degradation rate in average than the proteome background (single-tailed *t*-test, *P* = 0.029, tested for the null hypothesis that low EVI genes have no less degradation rates than the background genes) in HeLa cells (Fig 3E). As a negative control, the proteins encoded by the high TR outlier genes share similar degradation rates to the proteome background (single-tailed *t*-test, *P* = 0.10, tested for the null hypothesis that high TR genes have no less degradation rates than the background genes) (Fig 3F). This suggests that the translational pausing sites of the genes with slow elongation speeds may significantly facilitate the protein folding, thus increases their stability.

### Codon preference of low EVI genes in human cells

We next tried to assess the potential causal factor of the slow translation of the low EVI genes. Although the role of mRNA structure in affecting the elongation speed was under strong debate [16, 24, 25, 31], we tried to examine such an influence with our data. We calculated the mRNA fold energy per base for low-EVI and high-TR genes, respectively, using the MFOLD algorithm [46]. In each of the four cell lines, the low-EVI genes were not more stably folded than the high-TR genes (one-tailed KS-test, S10 Fig), showing that the mRNA structure stability did not play a significant role in slowing down the elongation. Thus, we deduct that the amino acid content, such as positively charged amino acids, may be a factor. We found that the fraction of positively charged amino acids (lysine, arginine and histidine, PCAAs) in low EVI genes was close to that in high TR genes, with the maximum difference less than 1.5%. In HBE and A549 cells, low EVI genes contained slightly more PCAAs than high TR genes, while *vice versa* in HeLa and H1299 cells (Table 1). In the 4 tested cell lines, we found that their low-EVI genes all had significantly different fraction of PCAAs than the genome average (Table 1). This indicated that the positive charge of the nascent peptides may have effects, but it is unlikely to be the primary factor to slow down the translation of low EVI genes, at least in the four analyzed cell lines of this study.

**Table 1:** Fraction of positively charged amino acids (lysine, arginine and histidine) in low EVI genes and high TR genes.

Cells	Fraction of positively charged amino acids			*P*-value (Two-tailed Chi-square test)	
	Low EVI genes	High TR genes	All genes	Low EVI vs. high TR genes	Low EVI vs. all genes
HeLa	13.7%	15.2%	14.3%	1.92x10^-12^	4.05x10^-8^
HBE	15.8%	14.8%		2.85x10^-5^	<10^−38^
A549	16.2%	14.6%		3.07x10^-8^	<10^−38^
H1299	16.5%	16.7%		0.462	<10^−38^

As a reference, the fraction of positively charged amino acids in all genes is 14.3% in all four analyzed cell lines.

We previously reported in bacteria that translation pausing sites are created by clustered slow-translating codons in the CDS that paired to low-abundance tRNA species [13, 14]. Here, we further tried to identify the slow-translating codons in these human cells that may serve as a causal factor of the translational pausing sites in the low EVI genes, respectively. For comparison across different cell lines, we quantified the codon preference by the Preference Score (PS) of low EVI genes (PS_Low-EVI_) in each cell line based on the RSCU of the low EVI genes *vs*. background genome. The higher the PS of a codon is, the more preferentially used in the low EVI genes than background genome.

**Fig 4:**
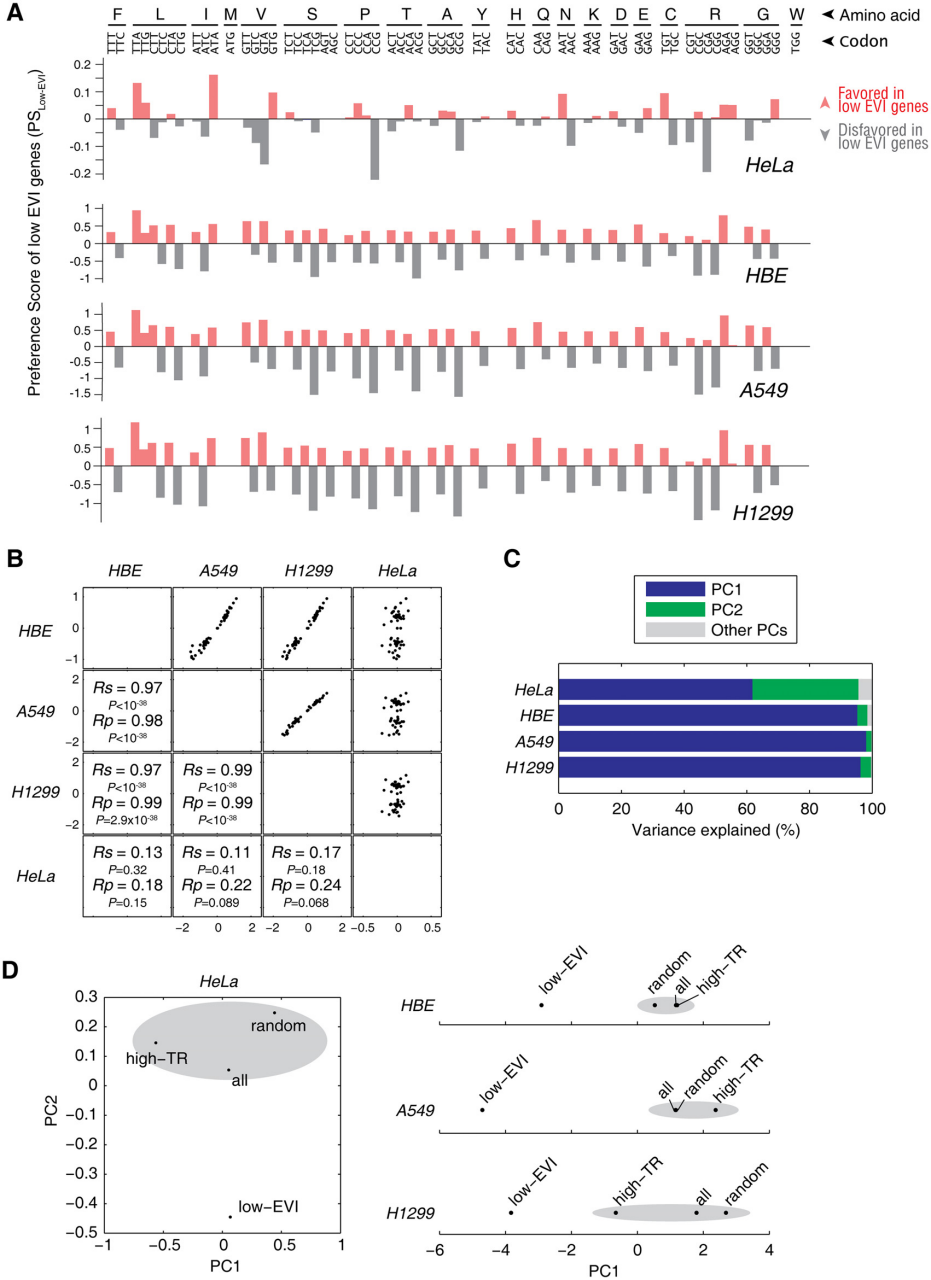
Codon preference of the slow-translating genes. (A) Codon preference of low EVI genes in the four cell lines based on Preference Score analysis. For details, please refer to Materials and Methods section. Favored and disfavored codons are shown in red and grey bars, respectively. (B) Mutual correlation of PS_Low-EVI_ of codons among all four analyzed cell lines. (C) Percentage of variance explained in the principle component analysis (PCA) on RSCU. (D) Principle component (PC) based clustering analysis. The PCs with the summed explained variance greater than 80% were taken for clustering analyses based on the standardized Euclidean distances. For each cell line, four groups of genes were analyzed. They were low EVI genes, high TR genes, all genes and a random subset of genes that were inside the 95% confidence ellipse (Fig 2A and 2B). The clusters with multiple gene groups are indicated by grey ellipses.

We found that the low EVI genes in all of the four tested cells showed a remarkable codon preference for most of the amino acids that was reflected by PS_Low-EVI_ (Fig 4A). In a general view, the composition of preferred and non-preferred codons in HeLa cells were different from the lung cells with a tissue-specific manner (Fig 4A). For example, the GGT and GGA codons that encodes glycine are disfavored in low EVI genes in HeLa cells (*P* = 0.03, Chi-squared test), but are favored in HBE, A549 and H1299 cells (*P* < 10^−38^ in all three cases). The GGG codon is favored in low EVI genes in HeLa cells (*P* = 0.02, Chi-squared test), but is disfavored in HBE, A549 and H1299 cells (*P* < 10^−38^ in all three cases) (Fig 4A). We also detected by the PS_Low-EVI_ analysis that certain synonymous codons are not necessarily and preferentially used. In HeLa cells, the three codons encoding isoleucine are very different in PS_Low-EVI_ values: ATA is highly preferred in the low EVI genes (*P* = 0.0003, Chi-squared test) (Fig 4A). In contrast, the codons encoding tyrosine and lysine showed minimal preference in the low EVI genes (Fig 4A).

To quantify the tissue-specific codon preference, we compared the codon PS_Low-EVI_ values among all of the tested cell lines (Fig 4B). The lung HBE, A549 and H1299 cells showed strong correlation to each other (*Rs* = 0.97~0.99), showing that they have significantly similar codon preference in their low EVI genes. On the contrary, the codon preference in HeLa cells are almost not correlated to the three lung-derived cells (*Rs* = 0.11~0.17, *P* = 0.18~0.41). These results confirmed the tissue-specific codon preference shown above.

To verify whether PS_Low-EVI_ can distinguish the low EVI genes in an unbiased manner, we examined the codon preference of four groups of genes in each cell line. They were low EVI genes, high TR genes, all genes, and a random subset of genes that locate inside the 99% confidence ellipse in the TR *vs*. EVI plots (Fig 1C and 1D). We performed principle component analysis (PCA) on the RSCU of all 61 sense codons in each cell and identified the principle component(s) (PC) that can collectively explain more than 80% of the variance (Fig 4C). In HeLa cells, the first two PCs explained 95.7% of the variance, while in the three lung-derived cells, the first PCs alone explained 95.2~98.1% of the variance, respectively (Fig 4C). We next performed hierarchical clustering with the distance metric of standardized Euclidean distance. Algorithm for computing distance between clusters was unweighed average distance (UPGMA). We found that low EVI genes were clustered as an independent group in 2-dimensional (for HeLa cells) or 1-dimensional (for HBE, A549 and H1299 cells) spaces, respectively (Fig 4D). Thus, the low EVI genes have distinct codon preference to slow down their translation in all of the cells examined in this study.

**Fig 5:**
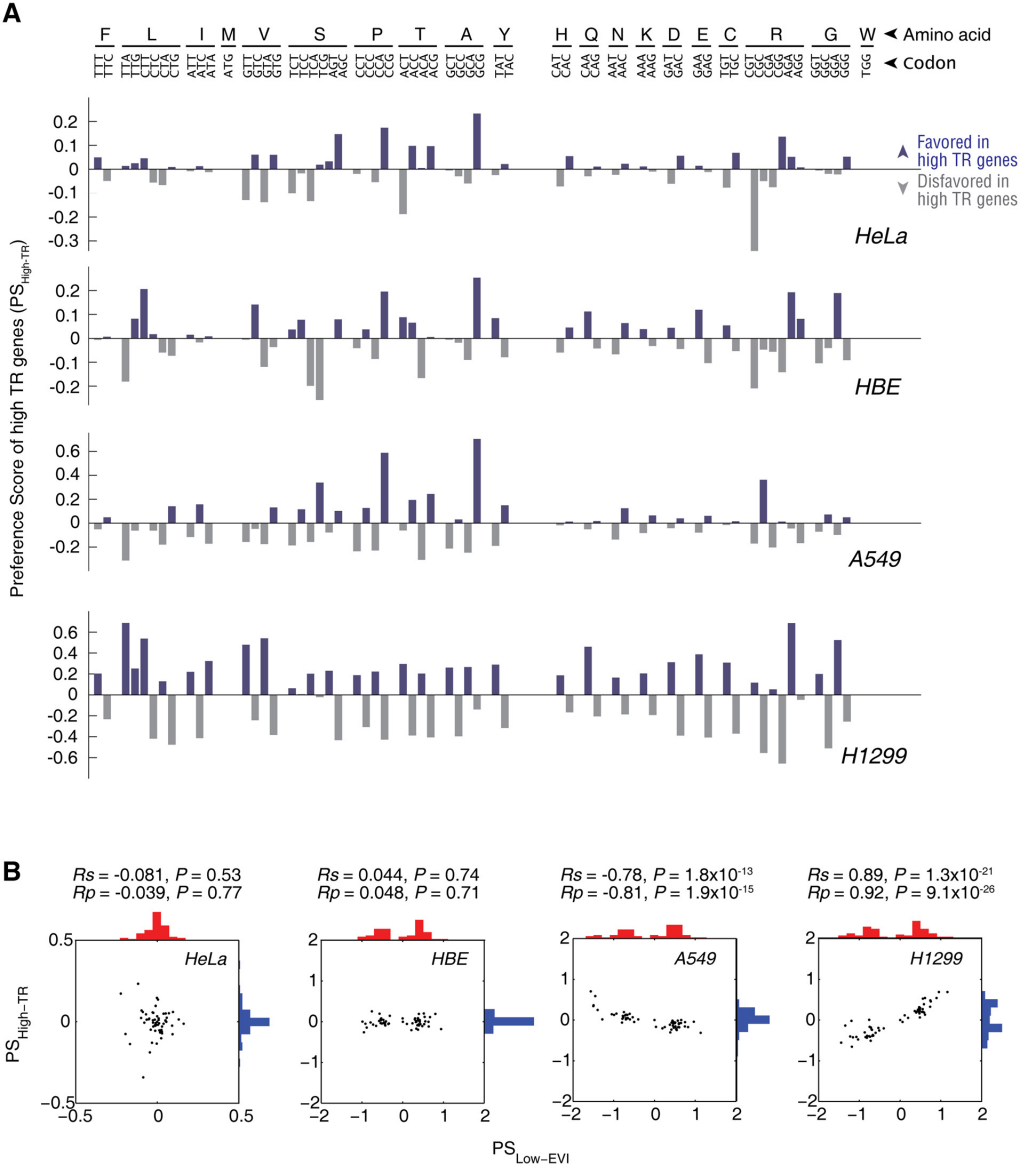
Codon preference of the high TR genes. (A) Preference Score of high TR genes (PS_High-TR_) of the four cell lines. (B) Correlation of the PS_High-TR_ and PS_Low-EVI_.

To further rule out the experimental and/or computational bias, we performed similar analyses on the codon preference of high TR genes (PS_High-TR_) (Fig 5A). The PS_High-TR_ is also cell-specific, indicating that the tRNA concentration is cell-specific either (S11 Fig). Since the high TR genes are translated faster, we expected a different codon preference of the high TR genes than low EVI genes. Indeed, in HeLa and HBE cells, the PS_High-TR_ and PS_Low-EVI_ have no significant correlation (*P* = 0.53~0.71, Fig 5B). We noted that PS_High-TR_ correlates to PS_Low-EVI_ in A549 cells (*Rs* = -0.78, Fig 5B) and H1299 cells (*Rs* = 0.89, Fig 5B), respectively; however, the distribution of PS_High-TR_ is significantly different from that of PS_Low-EVI_, with *P* = 6.99×10^−6^ and 2.20×10^−4^ in the two cell lines, respectively (KS-test on distribution).

Furthermore, we observed in all of tested cell lines that there was no significant correlations between PS (either PS_Low-EVI_ or PS_High-TR_) and codon usage (S12A Fig), tAI (S12B Fig) or mean codon elongation time (S13 Fig), respectively.

### Slow-translating genes and malignant phenotypes

We previously compared lung cancer A549 and H1299 cells with normal HBE cells, respectively, in terms of cancer phenotypes, translation initiation efficiency and proteome [6, 47]. To understand the biological relevance of EVI, we further examined with these cell lines to answer whether the slow-translating gene group is biased on regulating cancer-favorable phenotypes.

**Fig 6:**
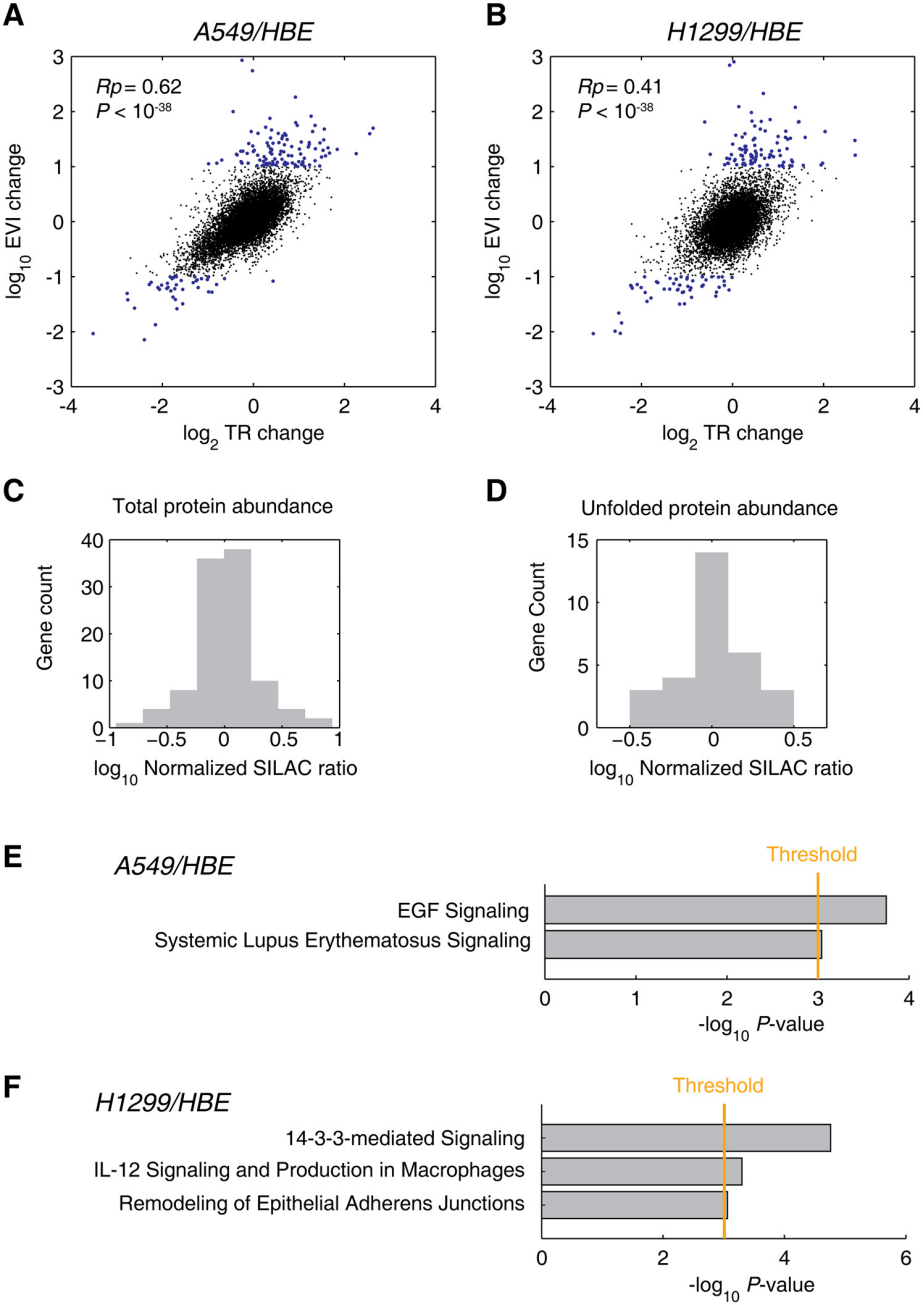
Relative EVI analysis and cancer relevance. (A, B) Correlation of the relative changes of TR and EVI comparing cancer with normal cells. A549/HBE (A) and H1299/HBE (B) results were shown, respectively; the genes with relative EVIs of greater than ±10 folds were indicated by blue dots. (C,D) Comparative proteomics analyses on the low-EVI genes comparing A549 cells with HBE cells. Relative abundance distributions of total proteins (C) and the unfolded proteins (D) are shown. (E, F) Top canonical pathway analysis of low relative EVIs by IPA. Low relative EVI genes (<10 folds) in A549/HBE (E) and H1299/HBE (F) comparisons were subjected to IPA analysis. The top canonical pathways with *P* < 0.001 (Fisher’s exact test provided by IPA indicated by the threshold line in orange) regulated by of these genes were shown respectively. The complete gene lists for IPA are included in the S2 Table with HGNC gene names.

First, we found that the TR change and EVI change correlate to each other in A549/HBE (*Rp* = 0.62; *P <* 10^−38^; Fig 6A) and H1299/HBE (*Rp* = 0.41; *P <* 10^−38^; Fig 6B) comparisons, respectively. This echoes the comparisons on the absolute TR and EVI changes (Fig 2B). However, no significant correlation between TR and EVI changes could be found for the EVI up-regulated genes, and only weak TR-EVI change correlation could be found for the EVI down-regulated genes (S14 Fig), indicating that the translation elongation speed is largely independently regulated for these genes.

Next, we tried to answer whether the elongation deceleration could significantly affect the protein abundance of these EVI down-regulated genes. Using our previously published comparative proteomics data [6], we found that the protein abundance of EVI down-regulated genes (A549 compared to HBE cell line) obeyed a normal-like distribution, with no significant difference from the proteome background (*P* = 0.82, two-tailed KS-test) (Fig 6C). Such results suggested that the protein quality of EVI down-regulated genes may be increased by slowing down elongation, which led to no significant impact on their protein abundance. In addition, we have recently reported that A549 cells have more abundant detergent-insoluble proteins than HBE cells [48], suggesting that lung cancer cells are exposed to more severe protein folding problems than normal cells in genome-wide scale. With the comparative proteomics data, we found that the relative abundance distribution of the EVI down-regulated gene product in the unfolded protein fraction had no significant difference comparing A549 with HBE cells (*P* = 0.85, two-tailed KS-test; Fig 6D). This result indicated that the EVI down-regulation may serve as an important factor to facilitate protein folding in cancer cells.

With IPA knowledgebase, we analyzed the genes with more than 10-fold down-regulation in their EVIs comparing lung cancer cells with normal cells (S5 Table). We found that A549 cells were decelerating the elongation of genes with low EVIs that is significantly relevant to maintain the EGF signaling pathway (*P* = 1.77×10^−4^; Fig 6E), which plays important roles in the proliferation and differentiation of lung cancer cells; in addition, systemic lupus erythematosus signaling was also significantly associated (*P* = 9.07×10^−4^; Fig 6E). The elongation speed regulation of H1299 cells focuses on the 14-3-3 (*P* = 1.73×10^−5^; Fig 6F; S15 Fig), IL-12 (*P* = 5.07×10^−4^; Fig 6F) and remodeling of epithelial adherens junctions (*P* = 8.76×10^−4^; Fig 6F). These pathways are known to be essential for the survival, active transcription and migrations of cancer cells as suggested by IPA. For example, the protein of 14-3-3 is highly active in lung and breast cancers interacting with AKT and MAPKs (S15 Fig). As a negative control, we found not significant pathways were enriched by analyzing the genes whose EVI were up-regulated for more than 10 folds in cancer cells.

On the contrary, we found that cancer cells tended to accelerate the translation elongation speed of tumor suppressor genes (TSGs). We took the identified TSGs in the TCGA lung adenocarcinoma datasets from TSGene 2.0 Database (http://bioinfo.mc.vanderbilt.edu/TSGene/download.cgi). The EVIs of these TSGs were elevated 1.89 and 1.75-fold in average in A549 and H1299 cells, respectively, as compared with HBE cells. Furthermore, these up-regulations were more significant in the more malignant H1299 cells (*P* = 0.02, single-tailed Mann-Whitney U-test against the genome-wide background) than in the less malignant A549 cells (*P* = 0.34, as above).

## Discussion

In this study, we realized the evaluation of global elongation speed at individual gene level for human cells under physiological conditions. Together with our previously defined TR analysis for translation initiation [6], we proposed a two-dimensional fine tuning of translational control to form a translation initiation preference on genes with high phenotype relevance, as well as to adjust translation elongation speed that is relevant to protein quality control. Mechanistically, we discerned the codon preference, especially the slow-translating codons, to explain the slowly translated genes in different human cell types. Therefore, we provided the genome-wide evidence, for the first time, to favor the importance of elongation speed deceleration for the maintenance of the malignant phenotypes of cancer cells at steady-state.

Indeed, using EVI to assess the translating speed is the basis and one of the central messages of this study. We tried to provide evidence at various levels, considering multiple independent factors to avoid biased conclusions. First, we demonstrated with RFP coverage analysis that the low EVI genes have significant translational pausing sites that locate mostly after 240 nt. Favorably, we previously found that most of folding-relevant translational pausing sites occur after 240 nt (~10kDa nascent peptide) in prokaryotes [13, 14]. A recent study reported similar phenomena in human cells under heat stress [44]. In addition, we generally ruled out the possible biases in translation initiation calculation caused by the 5’UTR regulatory elements, such as internal ribosomal entry site (IRES) and upstream open reading frames (uORF) [49]. These findings suggested that the EVI analysis in this study distinguished slow-translating genes in human cells. Second, we found that the low EVI genes are more stable in the degradation rate constant analysis. This finding is consistent with and reciprocally supports the pause-to-fold theories [13, 19] (reviewed in ref. [7, 20, 21]). Notably, our EVI evaluation provided genome-wide and individual gene resolution that moves the field forward. Third, as a negative control, we found that the most frequently initiated genes tend to have fast elongation speeds (Fig 2A and 2B). For example, the high TR genes *HIST1H1E* and *HIST1H4C* (S9A Fig) have been shown to have very stable and robust structures that are heat-resistant up to 60°C [50], implicating that the correct folding of these proteins may not be co-translational folding dependent. Favorably, O'Brien has proposed that faster translation of some proteins may assist their correct folding by speeding up termination [51].

In addition, we noted that no genes that have both high TR and low EVI properties. As an explanation, it is known that the high initiation efficiency and significant translational pausing sites will increase the risk of ribosome traffic jam [52] that frequently causes frame-shifting and premature drop-off [53], leading to aggregations of aberrant proteins. This is detrimental to cells by wasting ribosome resource, and thus should be maximally avoided per evolution, at least under physiological conditions, which has been evidenced by modeling and experiments [9, 54–56]. In addition, Chu et al have found that slow codons lead to postponed recycle of ribosomes that are the resources of next round translation initiation [57]. This serves as a reasonable explanation on the significant EVI-TR correlation demonstrated in our current study. These evolution relevant rationales biologically supported the validity of using EVI to evaluate elongation speed.

As a mechanistic explanation, we demonstrated the codon preference in terms of translation elongation speed in human cells. Accurate identifications of slow-translating codons relies on the quantitative and codon-wise tRNA abundance data; however, previous efforts found that such data are tissue-specific, time-dependent and difficult to obtain with anticodon-resolution in eukaryotic cells [58–60] (reviewed in ref. [20]). From the available tRNA data [58], we observed that the tRNA abundances between different tissues have general weak or insignificant correlation (|*R*|<0.4 or *P*>0.01) for most cases (S16 Fig). Interestingly, based on EVI analysis, we detected slow-translating codons that were tissue/cell type specific as well. With PCA, we found that one or two PC(s) would be sufficient to represent the codon preferences that were capable of distinguishing the low EVI genes from the background genomes. These results emphasized the validity of EVI examination and the codon preference in human cells. Notably, we observed no significant correlations between codon preference and codon usage or tAI, which was comparable with Ingolia *et al*, proposing that translation elongation speed was independent of codon usage for a remarkable proportion of genes [9].

As expected, we observed that EVI has very weak or no correlations with CAI or CBI, respectively; however, it is significantly correlated to Nc. This can be explained by the codon selection and translational elongation. Lower Nc values suggest that each amino acid is encoded by fewer codons, corresponding to higher homozygosity [42, 43]. In the extreme condition of the lowest Nc of 20, cells should not use the slow-translating codons for each amino acids in that consecutive slow-translating codons will remarkably prolong the translation duration and increase the risk of frameshift, ribosome drop-off and ribosome jamming [53, 61]. Indeed, this is maximally avoided in the real genomes [14]. Thus, the low Nc genes tend to use fast-translating codons, which coincides with their higher translation elongation speed measured in our study (higher EVI).

Indeed, the EVI measurement strategy shown in this study can be applied to more cell types and situations, which will lead to a complete resolution of identifying slow-translating codons in pan eukaryotic systems. Nevertheless, we realized that it is of great importance to independently validate this EVI evaluation on elongation speed by incorporating the tRNA-omics data into this computation per further technological breakthrough.

We demonstrated the possible relevance of co-translational folding to cancer phenotypes to justify the biological relevance of EVI as an example. In one way, as we previously reported, lung cancer cells have the loop-back enhancement of global translation initiation [6]. Hence, high TR should be a productivity-focused translational control mode. In the other way, to increase the quality of tumor-favorable proteins should be demanded by cancer cells. This was supported by the IPA and stability analyses on the slow-translating gene shown in this study. Specifically, EGF pathway over-activation, caused by such as EGFR mutation, has been identified as a prevalent mechanism for the onset and progression of lung cancers [62]. Interestingly, the low relative EVI genes in A549/HBE comparisons included the IPA clustered genes of p39, PI3K and SOS, which are all central messengers for the EGF pathway. Promoting the proper folding of these proteins in A549 cells should be favorable for their malignant phenotypes. Supportive to this notion, similar evidence was also found in the H1299/HBE comparisons.

We realized that the above rationale could not lead to definitive conclusion to link co-translational folding to cancer. But, the significant acceleration of TSG translation elongation especially in highly malignant cancer cells is an interesting finding in this study. Due to the suppression of translational pausing, the multi-domain TSGs would probably become less folded, thus disables their functions. This facilitates the malignancy of the cancer cells. In the contrast, some other genes were decelerated meanwhile for the same purpose. For example, 14-3-3 in H1299 cells is one of the most decelerated genes. 14-3-3 interacts with numerous misfolded proteins and intrinsic disordered proteins and thus plays a key role in aggresome formation [63, 64], helping to clear up the misfolded proteins generated in lung cancer cells, including the non-functional and misfolded TSGs. It also regulates functions of hundreds of proteins by conformational modulation (reviewed in [65]) and involves in cancer development, progression and chemoresistance (reviewed in [66, 67]). Therefore, the proper folding of 14-3-3 is crucial for the malignancy, and the cancer cells consolidated the folding by relatively decelerating 14-3-3 translation elongation. To be noted, we demonstrated that the deceleration of elongation did not statistically change the protein abundance of the EVI down-regulated genes, and stabilizes the protein folding in an environment in which protein misfolding occurs more frequently. These implicate that the protein quality of their gene products may be increased. Although further studies are necessary to fully reveal the detailed mechanisms, our EVI evaluation shed lights on the translational elongation regulation and its synergy with translational initiation regulation in cancer.

## Methods

### Cell culture

HeLa cells (ATCC, Rockville, MD) were maintained in DMEM (Invitrogen) supplemented with 10% fetal bovine serum (PAA), 1% penicillin/streptomycin and 10 μg/ml ciprofloxacin. Normal human bronchial epithelial (HBE) cells as well as human lung cancer A549 and H1299 cells were maintained as described previously [6]. In brief, the cells were maintained in Dulbecco’s modified Eagle’s medium (Invitrogen, Carlsbad, CA), supplemented with 10% fetal bovine serum (PAA Australia, Weike Biochemical Reagent, Shanghai, China), 1% penicillin/streptomycin and 10 μg/mL ciprofloxacin.

### Transcriptome and full length translating mRNA sequencing

The total RNA and RNC-RNA extraction from HeLa cells was performed as previously described [6]. A pooled sample from 3 independent experiments was used for subsequent RNA-seq regarding mRNA and RNC-RNA, respectively. For mRNA and RNC-mRNA, the polyA+ mRNA was selected by RNA Purification Beads (Illumina). The library was constructed by using the Illumina TruSeq RNA sample Prep Kit v2 and sequenced by the Illumina HiSeq-2000 for 50 cycles. High quality reads that passed the Illumina quality filters were kept for the sequence analysis. These sequencing datasets are available at Gene Expression Omnibus database (accession number GSE46613).

### Ribosome profiling

It has been debated that the application of cycloheximide in ribosome profiling may cause artifact like the excessive accumulation of RFPs near the start codons [9]. However, recent studies confirmed that the cycloheximide gives comparable result as the no-drug protocol when properly processed [68]. Brief treatment of cells by cycloheximide does not distort the ribosome profiling measurements [9]. In addition, elongation inhibitors like cycloheximide are especially useful to preserve the translational state during sample preparation [9, 69]. Therefore, we chose to use cycloheximide in our ribosome profiling experiment.

Cells were pre-treated with 100 mg/ml cycloheximide for 15 min, followed by 3 washes with pre-chilled phosphate buffered saline (PBS) prior to the addition of 2 ml cell lysis buffer [1% Triton X-100 in ribosome buffer (RB buffer, 20 mM HEPES-KOH (pH 7.4), 15 mM MgCl_2_, 200 mM KCl, 100 μg/ml cycloheximide and 2mM dithiothreitol)] to each of the T-75 flasks. After 30-min ice-bath, cell lysates were split into 2 pre-chilled 1.5 ml tubes. Cell debris was removed by centrifugation at 16,200 ×*g* for 10 min at 4°C. Supernatants were transferred into new pre-chilled 1.5 ml tubes with addition of 2 μl Ribolock RNase Inhibitor (40U/μl, Fermentas) in each tube. RNase I (10U/μl, Fermentas) was then added at 0.2 μl per tube, followed by incubation at 37°C for 15min and reaction termination with 1% SDS (1/10 volume per tube). The digested samples were pooled and layered on the surface of 15 ml sucrose buffer (30% sucrose in RB buffer). The ribosomes were pelleted by ultracentrifugation at 185,000 ×*g* for 5 h at 4°C. RNA extraction was then performed by Trizol method and ribosomal RNA (rRNA) was depleted using Ribo-Zero rRNA Removal Kit (Human/Mouse/Rat) (Epicenter) by following the manufacturer’s instructions. The ~28nt RNA fragments were considered as ribosome footprints (RFPs), which can be visualized in the RFP samples, while are non-detectable in the intact RNC-RNA samples.

The sequencing libraries of RFP were constructed, following the NEBNext Multiplex Small RNA Library Prep Set for Illumina Guide (NEB). The library was resolved by a 6% polyacrylamide gel. The fraction with the insertion size ~28nt was excised and purified from the gel. This fraction was sequenced by an Illumina HiSeq-2000 sequencer for 36 or 50 cycles. The sequencing datasets for HeLa, HBE, A549 and H1299 cells are available at Gene Expression Omnibus database (accession number GSE46613, reviewer access link: http://www.ncbi.nlm.nih.gov/geo/query/acc.cgi?token=tfadpuykakkowxi&acc=GSE46613).

We analyzed the read density near the start codon according to the method described in [9]. All 4 cell lines in our study showed the peak of normalized average reads after the start codons are less than 2 (S17 Fig), comparable to the no-drug control in [9]. This evidenced that the cycloheximide in our study did not create artifacts in ribosome profiles.

### Sequence analysis

For ribosome profiling datasets, the adapter sequences were removed from all reads. Reads were truncated at their first nucleotides, whose Phred quality scores were less than 10. Reads shorter than 18 nt were then discarded. The rest high quality reads were aligned to the RefSeq-RNA reference sequence (downloaded from http://hgdownload.cse.ucsc.edu/downloads, accessed on Jan. 21st, 2013) using FANSe 2 algorithm [70] with the parameters –L60 –E2 –U1 –S10. For mRNA and RNC-mRNA sequencing datasets, the reads were mapped to RefSeq-RNA reference sequence with FANSe2 algorithm with the parameters #x2013;L55 –E4 –U0 –S10. Alternative splice variants were merged [6]. The expression levels were estimated by using the rpkM unit [71]. The mRNA length information was also acquired from RefSeq.

### Hotelling's T^2^ ellipse analysis

All genes were located in the TR against EVI scatter plot. Principle component analysis followed by multivariate generalization of t-test was performed to compute the Hotelling's T2 statistics [72]. The 99% confidence ellipse was calculated according to Li et al [73]. In brief, for bivariate observation values Z with a sample size of *n*, considering its unbiased estimate of covariance matrix S, a (1-α) confidence ellipse for prediction is given by the equation

$$\left(Z-\bar{Z}{)}^{\prime }S^{-1}(Z-\bar{Z})=\frac{2(n+1)(n-1)}{n(n-2)}F_{2,n-2}(1-\alpha \right)$$

Where *F*_2,n-2_(1-α) is the (1-α) critical value of an *F* variate with degrees of freedom 2 and *n*-2.

### Codon preference analysis

The synonymous codon selection preference was calculated using the method of relative synonymous codon usage (RSCU) [74]:

$$RSCU_{ij}=\frac{x_{ij}}{\frac{1}{n_{i}}\sum_{j=1}^{n_{i}}x_{ij}}$$

where *x*_*ij*_ is the usage of the *j*-th codon for the *i*-th amino acid, which is encoded by *n*_*i*_ codons [74].

The Preference Score (PS) for each codon of a certain group of genes was defined as the RSCU of this codon in this group of genes *vs*. that in the all genes, in log2 scale: $PS={\text{log}}_{2}(RSCU_{\text{gene group}}/RSCU_{\text{all}})$. A positive value of PS denotes favored codon in this gene group, while a negative value indicates a disfavored codon for such genes.

The CAI of a gene was calculated according to Sharp *et al*. [27]: $CAI=\text{exp}\left(\frac{1}{L}\sum_{i=1}^{L}\text{log}\frac{f_{i}}{\text{max}(f_{j})}\right)$, where L is the number of codons, *f*_i_ is the frequency of the codon i, and max(*f*_j_) is the maximum codon frequency for that amino acid.

The CBI was calculated according to Bennetzen *et al*. [41]: $CBI=\frac{N_{pfr}-N_{ran}}{N_{tot}-N_{ran}}$, where *N*_*pfr*_ is the total number of occurrences of preferred codons, *N*_*ran*_ is the expected number of the preferred codons if all synonymous codons were used equally, and *N*_*tot*_ is the total number of the 17 amino acids encoded by the preferred codons.

The effective number of codons (Nc) was calculated according to Wright *et al*. [42]: $Nc=2+\frac{9}{F_{2}}+\frac{1}{F_{3}}+\frac{5}{F_{4}}+\frac{3}{F_{6}}$, where *F*_*n*_ is the average homozygosity for the amino acids having a degeneracy of *n* codons.

The tAI of each codon was calculated according to dos Reis *et al*. [75].

### Ingenuity Pathway Analysis (IPA)

The genes with low relative EVIs and their respective absolute number of relative fold changes were uploaded and analyzed by IPA (http://www.ingenuity.com/). Core analysis of IPA was then performed as we previously reported, [6, 76]. The likelihood of associated between a set of genes with a pathway in Global Functional Analysis (GFA) and Global Canonical Pathways (GCP) was measured by the *P*-value, calculated by using the right-tailed Fisher’s Exact Test. *P*<0.001 was considered statistically significant.

## Supporting Information

S1 Table List of the TR and EVI values of all of the tested cells. (XLSX)

S2 Table List of gene quantifications of mRNA-seq, RNC-seq and Ribo-seq in HeLa, HBE, A549 and H1299 cells. Splice variants were merged and HGNC gene names were provided.
(XLSX)

S3 Table The threshold of the top 1% of TR and the lowest 1% of EVI. (DOCX)

S4 Table Overlapping low-EVI and high-TR genes among different cell lines. (DOCX)

S5 Table List of genes with the EVI change of more than 10 folds, comparing A549 and H1299 with HBE cells, respectively. (XLSX)

S1 Fig The distribution of RNC-mRNA ribosome densities (D = TR/EVI). (A) D distribution in each analyzed cell line. (B) D correlations between cell lines. *Rs* = Spearman *R*.
(PDF)

S2 Fig The gene distribution based on TR or EVI. (A) Empirical cumulative density function (CDF). The grey dashed line denotes the top 1% of TR or the lowest 1% of EVI. The threshold values are listed in the S2 Table. (B) Empirical probability density function (PDF).
(PDF)

S3 Fig The EVI calculated excluding 5’-UTR (x-axes) and the EVI calculated including 5’-UTR (y-axes) in all four tested cell lines. *Rp* = Pearson *R*; *Rs* = Spearman *R*.
(PDF)

S4 Fig The mutual correlation of gene expression levels of the 4 analyzed cell lines at the mRNA (A), RNC-mRNA (B) and RFP (C) levels. The Spearman correlation coefficient (*Rs*), Pearson correlation coefficient (*Rp*) and their corresponding *P*-values are indicated the Figs (D-F).
(PDF)

S5 Fig The Gene ontology enrichment analysis (Biological Processes, BP) for the low-EVI genes in the four cell lines. The analysis was performed using PANTHER website (http://pantherdb.org/). The significance level was set to *P*<0.01.
(PDF)

S6 Fig Correlation between EVI and codon adaptation index (CAI). The *Rs* and its *p*-value are shown on the top of each panel.
(PDF)

S7 Fig Correlation between EVI and codon bias index (CBI). The *Rs*, *Rp* and their *P*-values are indicated on the top of each panel.
(PDF)

S8 Fig Correlation between EVI and Effective Number of Codons (Nc). The *Rs*, *Rp* and their *P*-values are shown on the top of each panel.
(PDF)

S9 Fig The RFP coverage of representative genes with high TR (blue) and low EVI (red) genes in HeLa (A), HBE (B), A549 (C) and H1299 (D) cells, respectively. The sequence coverage range of each plot is indicated in the parentheses above each mRNA illustrations. The full length mRNAs are shown in thin lines, and the corresponding CDS regions are marked with thick lines.
(PDF)

S10 Fig The mRNA folding energy of the low-EVI and high-TR genes in four cell lines, calculated using MFOLD algorithm. One-tailed KS-test were performed for each cell line under the null hypothesize that the low-EVI genes are not more stably folded than the high-TR genes.
(PDF)

S11 Fig Plot matrix of the PS_High-TR_. The *Rs*, *Rp* and their *P*-values are indicated.
(PDF)

S12 Fig The PS_Low-EVI_ and PS_High-TR_ versus codon usage (A) and tAI (B) in HeLa, HBE, A549 and H1299 cells, respectively. The *Rp*, *Rs* and their *P*-values (*Pp* and *Ps*) are listed in the tables.
(PDF)

S13 Fig Correlation between PS_Low-EVI_ (calculated in this study) and mean codon elongation time for HeLa cells. (PDF)

S14 Fig The EVI-TR correlation of the EVI up- and down-regulated genes in A549 and H1299 cells, respectively. The Spearman *R* (*Rs*) and the *P*-values (*Ps*) were indicated.
(PDF)

S15 Fig The top canonical pathway, 14-3-3 mediated signaling pathway, of the slow-translating genes, comparing lung cancer cells H1299 against the normal lung cells HBE. (PDF)

S16 Fig Mutual correlation of tRNA content of different human tissues. (A) Plot matrix of mutual tRNA correlation of 7 human tissues normalized by the brain tRNA. (B) *Rp* and the –log10 p-values of the plot matrix. (C) The *Rs* and the #x2013;log10 p-values of the plot matrix. For (B,C), the numbers on the axes represent the tissues: 1 = liver, 2 = vulva, 3 = testis, 4 = ovary, 5 = thymus, 6 = lymph node, 7 = spleen.
(PDF)

S17 Fig Metagene analysis of translation initiation of the 4 tested cell lines. Average ribosome read density profiles of all well-expressed genes with at least 200 RFP reads are shown plotted.
(PDF)
