# Supplementary material for: Genome-Wide and Experimental Resolution of Relative Translation Elongation Speed at Individual Gene Level in Human Cells
Source: PLoS Genet. 2016 Feb 29;12(2):e1005901. doi: 10.1371/journal.pgen.1005901 (PMC4771717; doi:10.1371/journal.pgen.1005901)
Supplement: S3 Table — (DOCX) [file pgen.1005901.s003.docx]

**Table S3.** The threshold of the top 1% of TR and the lowest 1% of EVI.

| Cell | HeLa | HBE | A549 | H1299 |
| --- | --- | --- | --- | --- |
| Top 1% of TR | 2.138704 | 1.956761 | 2.20135 | 1.995767 |
| Lowest 1% of EVI | -0.92639 | -1.58485 | -2.17655 | -1.69894 |
