# Supplementary material for: Genome-Wide and Experimental Resolution of Relative Translation Elongation Speed at Individual Gene Level in Human Cells
Source: PLoS Genet. 2016 Feb 29;12(2):e1005901. doi: 10.1371/journal.pgen.1005901 (PMC4771717; doi:10.1371/journal.pgen.1005901)
Supplement: S4 Table — (DOCX) [file pgen.1005901.s004.docx]

**Table S4:** Overlapping low-EVI and high-TR genes among different cell lines.

1. 49 overlapping low-EVI genes among the three analyzed lung-derived cell lines:

*APC, ATRX, SMC4, UACA, VPS13A, VPS13C, RGPD2, PHF21A, CCDC88A, SMC6, SGOL2, GOLGA4, RIF1, MTRNR2L1, MTRNR2L3, MTRNR2L6, MTRNR2L8, MTRNR2L10, ASPM, ANKRD26, GOLGB1, CHD1, DST, ARID4A, TPR, HIST1H2AM, HIST1H4C, TRIP11, NEMF, PPIG, RAD50, AKAP9, TMF1, UTRN, BAZ2B, CEP350, KIF14, ZNF292, ZC3H13, SMC5, KIF20B, CENPF, MIS18BP1, MTBP, CTC1, HINT3, BOD1L1, GCC2, BMP8A*

1. 4 overlapping high-TR genes among the 3 analyzed lung-derived cell lines:

*NDUFB3, COX6C, TM2D1, ZNF564*

1. 4 overlapping low-EVI genes among all 4 analyzed cell lines:

*PHF21A, UTRN, MTBP, CTC1*
