# Supplementary material for: Genome-Wide and Experimental Resolution of Relative Translation Elongation Speed at Individual Gene Level in Human Cells
Source: PLoS Genet. 2016 Feb 29;12(2):e1005901. doi: 10.1371/journal.pgen.1005901 (PMC4771717; doi:10.1371/journal.pgen.1005901)
Supplement: S1 Fig — (A) D distribution in each analyzed cell line. (B) D correlations between cell lines. Rs = Spearman R. (PDF) [file pgen.1005901.s006.pdf]

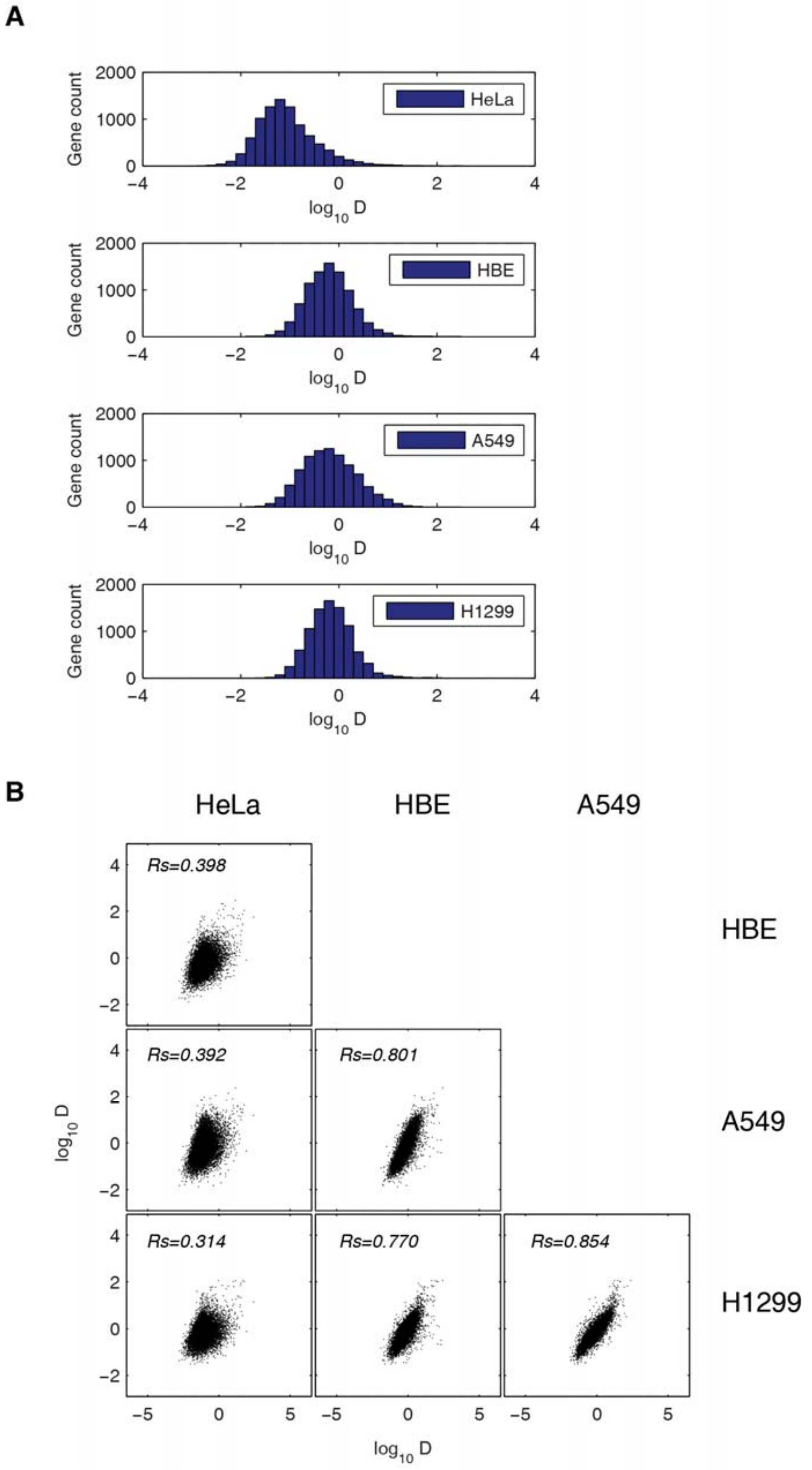

Figure S1: The distribution of RNC-mRNA ribosome densities (D=TR/EVI). (A) D distribution in each analyzed cell line. (B) D correlations between cell lines. *Rs* = Spearman *R*.
