# Supplementary material for: Genome-Wide and Experimental Resolution of Relative Translation Elongation Speed at Individual Gene Level in Human Cells
Source: PLoS Genet. 2016 Feb 29;12(2):e1005901. doi: 10.1371/journal.pgen.1005901 (PMC4771717; doi:10.1371/journal.pgen.1005901)
Supplement: S2 Fig — (A) Empirical cumulative density function (CDF). The grey dashed line denotes the top 1% of TR or the lowest 1% of EVI. The threshold values are listed in the S2 Table. (B) Empirical probability density function (PDF). (PDF) [file pgen.1005901.s007.pdf]

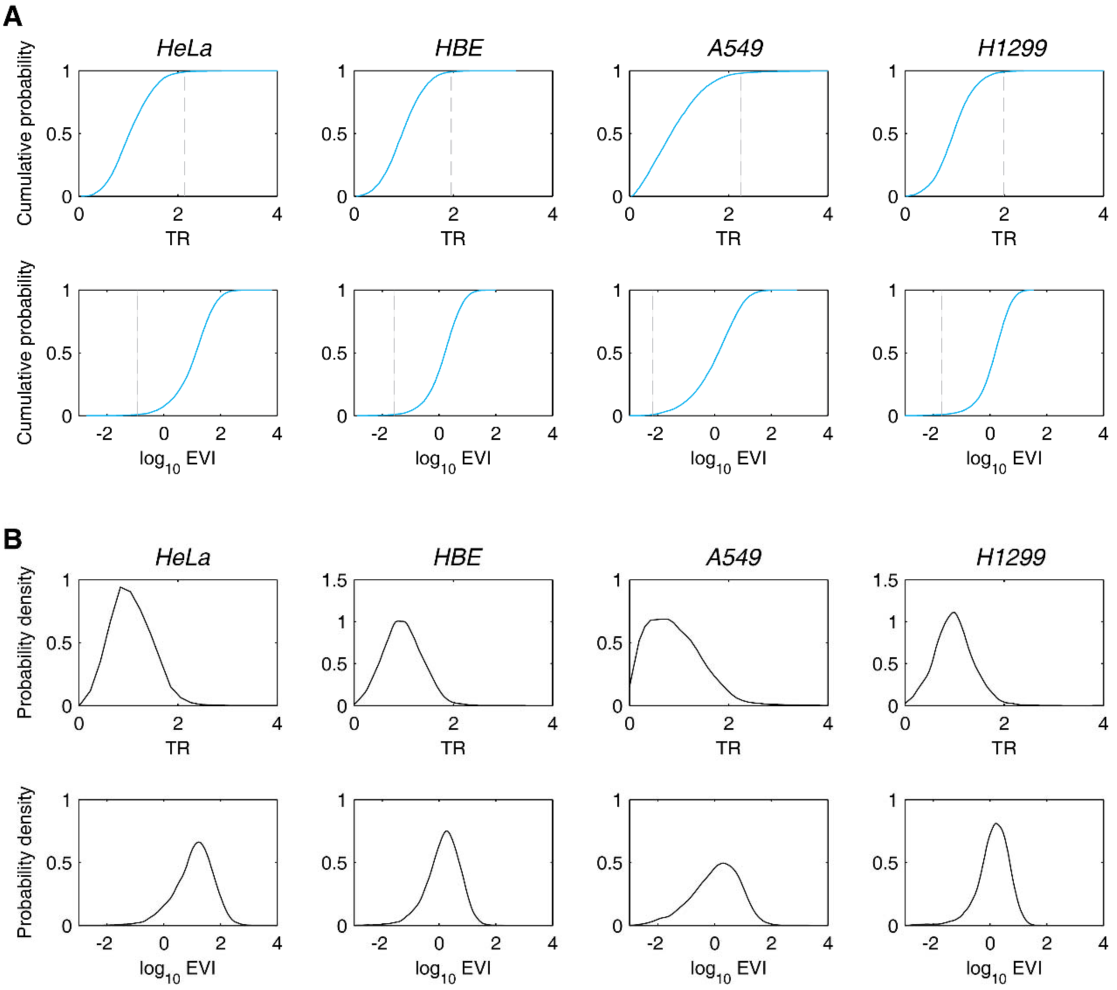

Figure S2: The gene distribution based on TR or EVI. (A) Empirical cumulative density function (CDF). The grey dashed line denotes the top 1% of TR or the lowest 1% of EVI. The threshold values are listed in the Supplementary Table S2. (B) Empirical probability density function (PDF).
