# Supplementary material for: Genome-Wide and Experimental Resolution of Relative Translation Elongation Speed at Individual Gene Level in Human Cells
Source: PLoS Genet. 2016 Feb 29;12(2):e1005901. doi: 10.1371/journal.pgen.1005901 (PMC4771717; doi:10.1371/journal.pgen.1005901)
Supplement: S3 Fig — Rp = Pearson R; Rs = Spearman R. (PDF) [file pgen.1005901.s008.pdf]

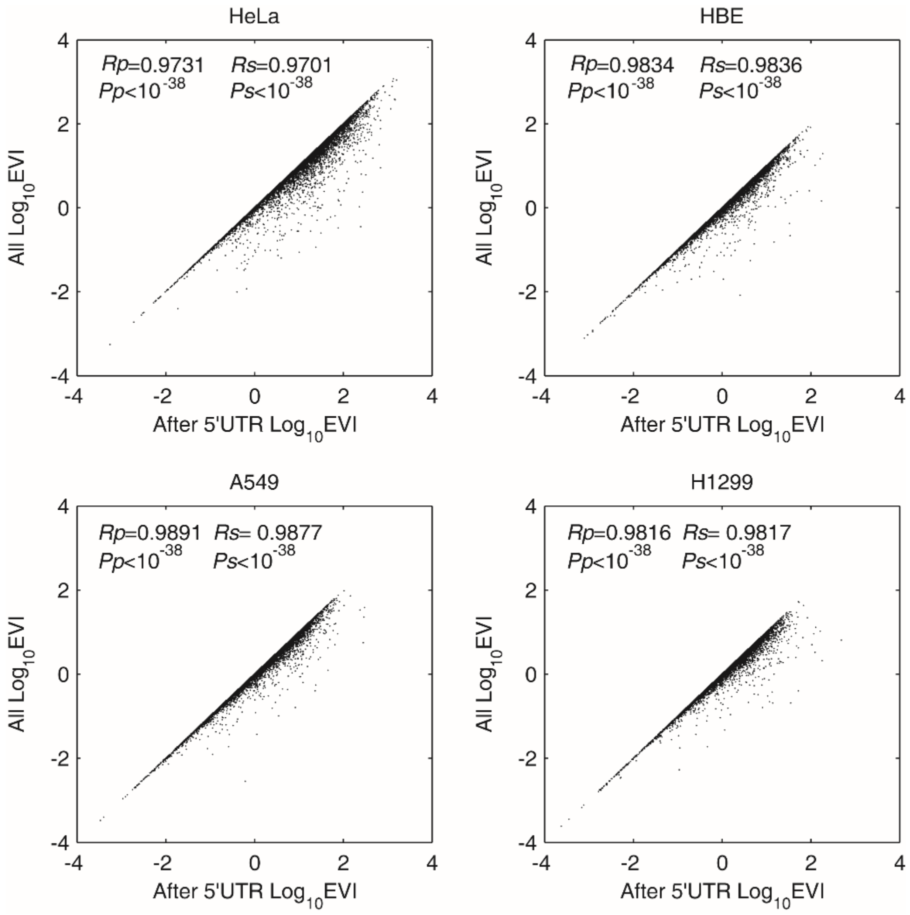

Figure S3: The EVI calculated excluding 5’-UTR (x-axes) and the EVI calculated including 5’-UTR (y-axes) in all four tested cell lines. *Rp* = Pearson *R*; *Rs* = Spearman *R*.
