# Supplementary material for: Genome-Wide and Experimental Resolution of Relative Translation Elongation Speed at Individual Gene Level in Human Cells
Source: PLoS Genet. 2016 Feb 29;12(2):e1005901. doi: 10.1371/journal.pgen.1005901 (PMC4771717; doi:10.1371/journal.pgen.1005901)
Supplement: S4 Fig — The Spearman correlation coefficient (Rs), Pearson correlation coefficient (Rp) and their corresponding P-values are indicated the Figs (D-F). (PDF) [file pgen.1005901.s009.pdf]

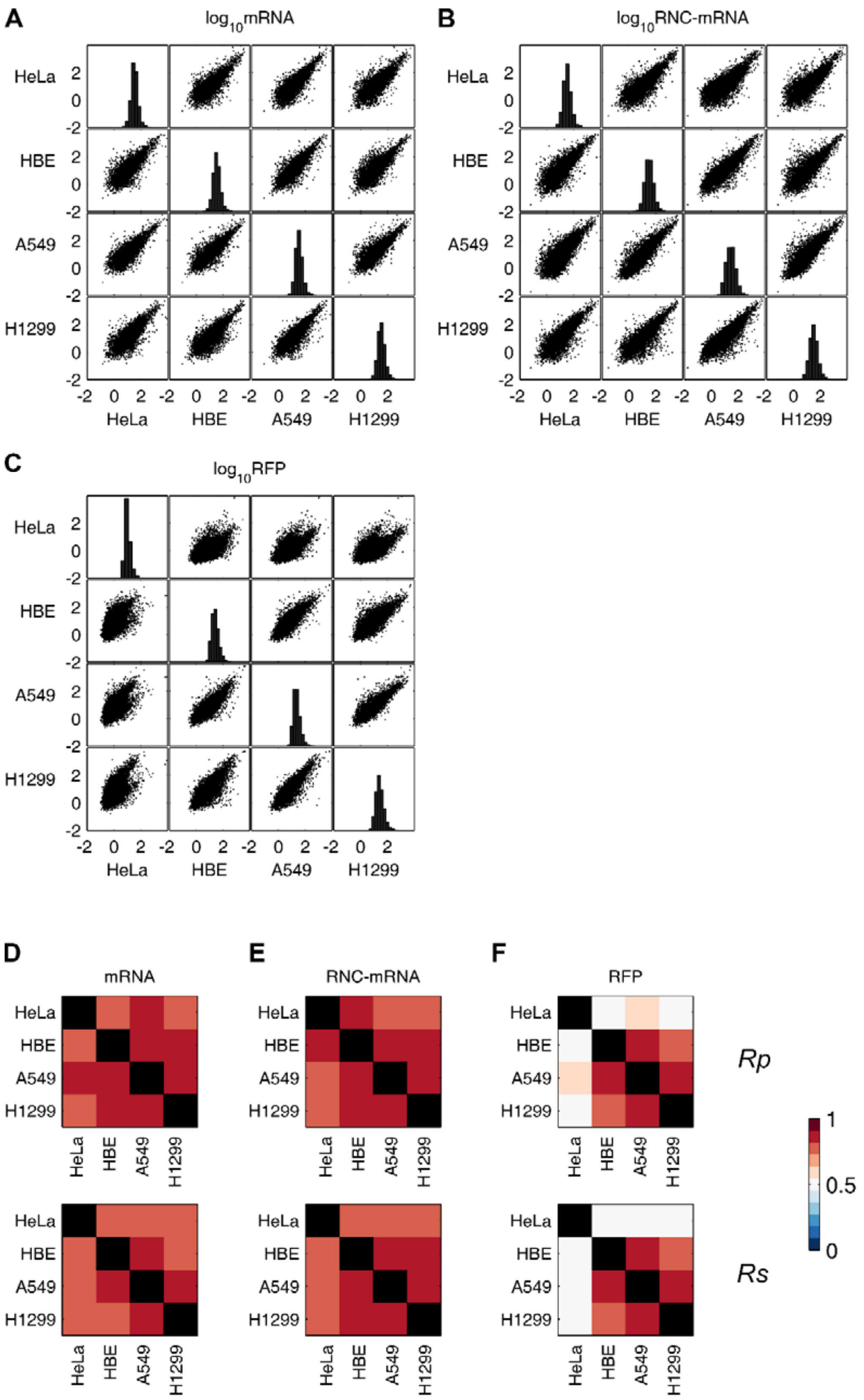

Figure S4: The mutual correlation of gene expression levels of the 4 analyzed cell lines at the mRNA (A), RNC-mRNA (B) and RFP (C) levels. The Spearman correlation coefficient (*Rs*), Pearson correlation coefficient (*Rp*) and their corresponding *P*-values are indicated the figures (D-F).
