# Supplementary material for: Genome-Wide and Experimental Resolution of Relative Translation Elongation Speed at Individual Gene Level in Human Cells
Source: PLoS Genet. 2016 Feb 29;12(2):e1005901. doi: 10.1371/journal.pgen.1005901 (PMC4771717; doi:10.1371/journal.pgen.1005901)
Supplement: S5 Fig — The analysis was performed using PANTHER website (http://pantherdb.org/). The significance level was set to P<0.01. (PDF) [file pgen.1005901.s010.pdf]

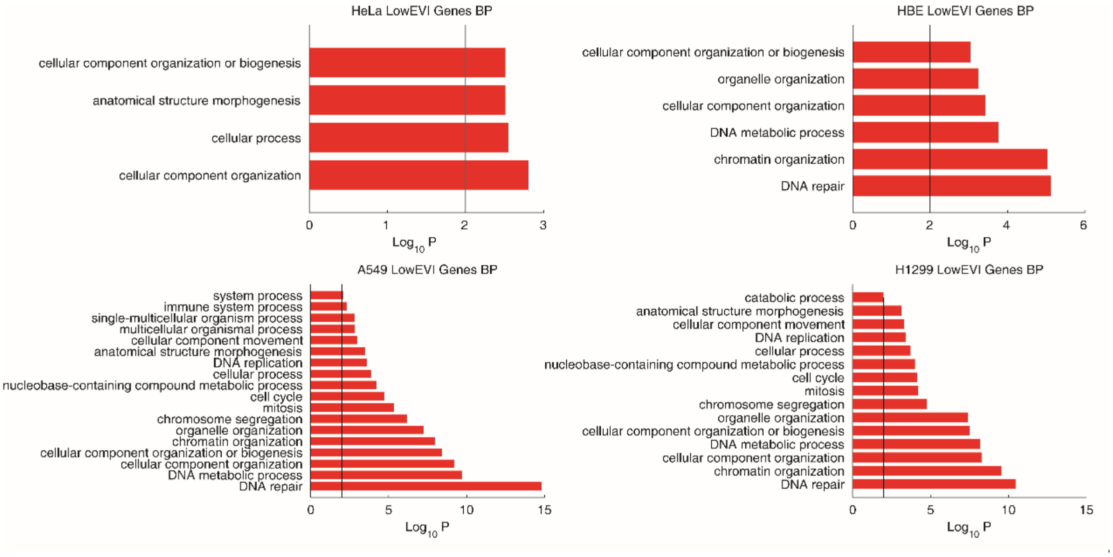

Figure S5: The Gene ontology enrichment analysis (Biological Processes, BP) for the low-EVI genes in the four cell lines. The analysis was performed using PANTHER website (http://pantherdb.org/) [1]. The significance level was set to *P*<0.01.

1. Mi, H., A. Muruganujan, and P.D. Thomas, *PANTHER in 2013: modeling the evolution of gene function, and other gene attributes, in the context of phylogenetic trees.* Nucleic Acids Res, 2013. **41**(Database issue): p. D377-86.
