# Supplementary material for: Genome-Wide and Experimental Resolution of Relative Translation Elongation Speed at Individual Gene Level in Human Cells
Source: PLoS Genet. 2016 Feb 29;12(2):e1005901. doi: 10.1371/journal.pgen.1005901 (PMC4771717; doi:10.1371/journal.pgen.1005901)
Supplement: S6 Fig — The Rs and its p-value are shown on the top of each panel. (PDF) [file pgen.1005901.s011.pdf]

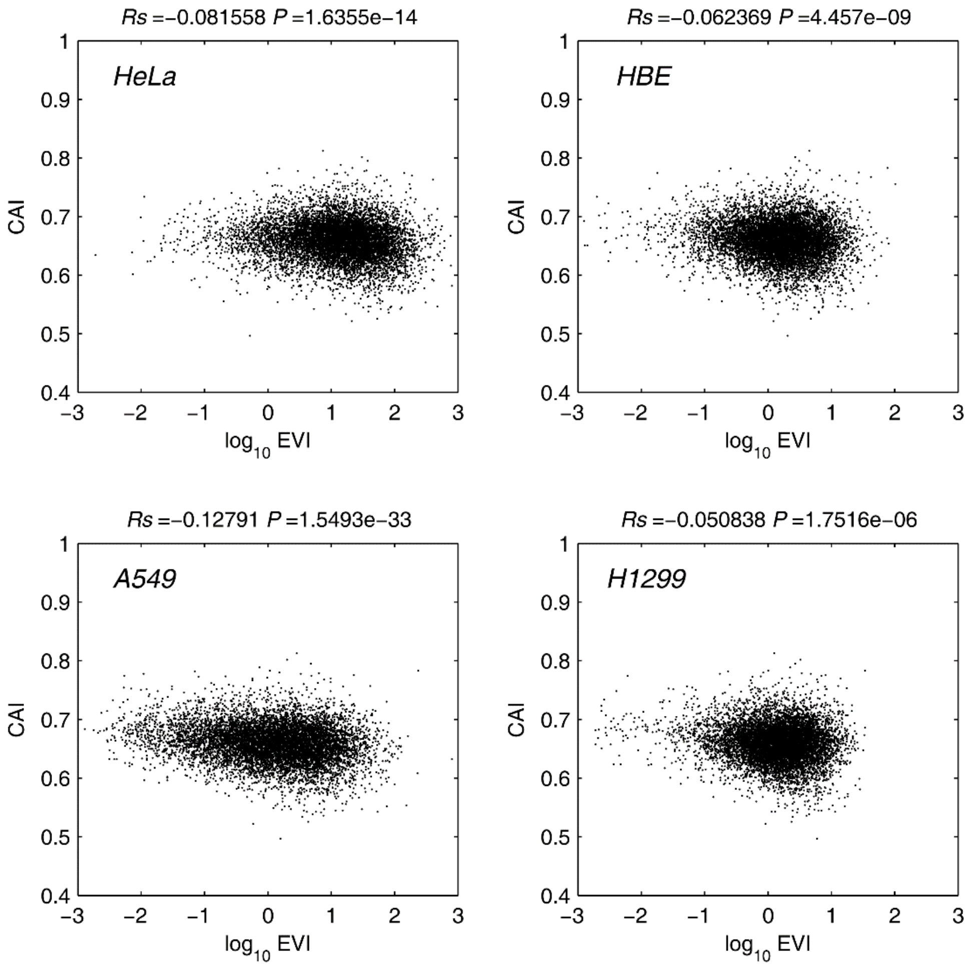

Figure S6: Correlation between EVI and codon adaptation index (CAI). CAI values for the genes were calculated according to [1]. The *Rs* and its *p*-value are shown on the top of each panel.

1. Sharp, P.M. and W.H. Li, *The codon Adaptation Index--a measure of directional synonymous codon usage bias, and its potential applications.* Nucleic Acids Res, 1987. **15**(3): p. 1281-95.
