# Supplementary material for: Genome-Wide and Experimental Resolution of Relative Translation Elongation Speed at Individual Gene Level in Human Cells
Source: PLoS Genet. 2016 Feb 29;12(2):e1005901. doi: 10.1371/journal.pgen.1005901 (PMC4771717; doi:10.1371/journal.pgen.1005901)
Supplement: S7 Fig — The Rs, Rp and their P-values are indicated on the top of each panel. (PDF) [file pgen.1005901.s012.pdf]

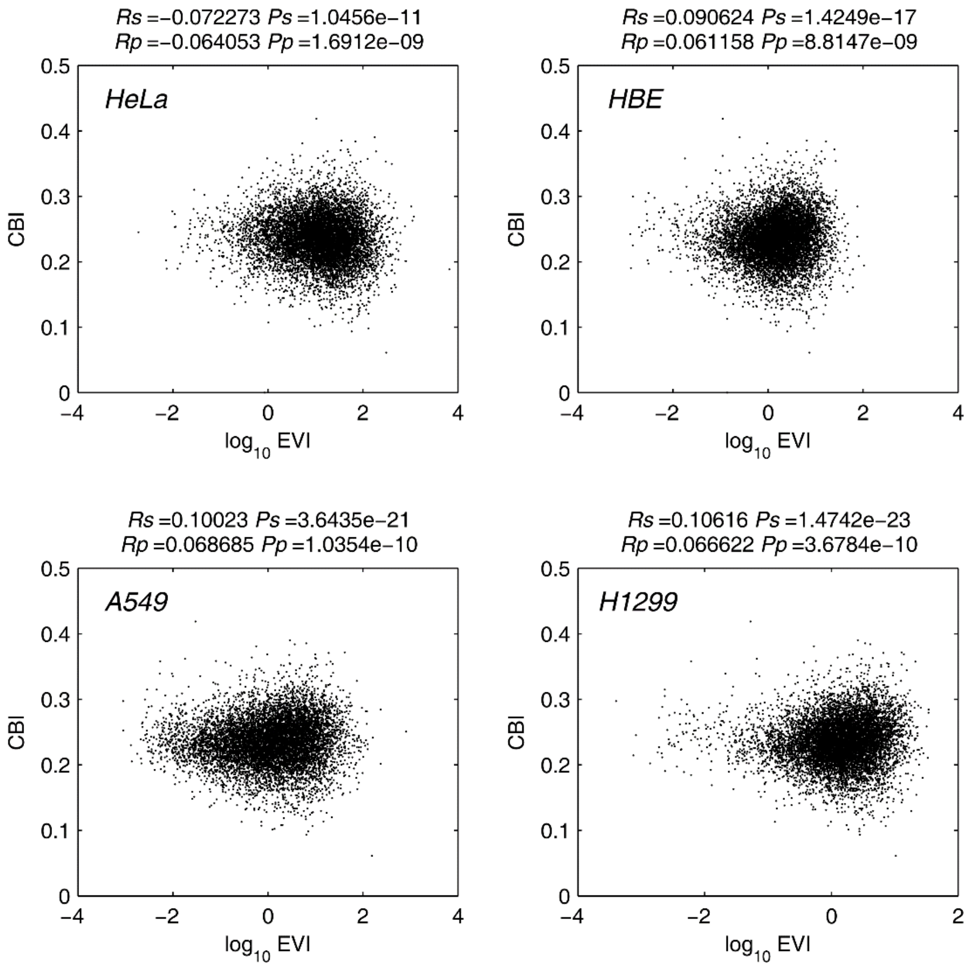

Figure S7: Correlation between EVI and codon bias index (CBI). CBI values for the genes were calculated according to [1]. The *Rs*, *Rp* and their *P*-values are indicated on the top of each panel.

1. Bennetzen, J.L. and B.D. Hall, *Codon selection in yeast.* J Biol Chem, 1982. **257**(6): p. 3026-31.
