# Supplementary material for: Genome-Wide and Experimental Resolution of Relative Translation Elongation Speed at Individual Gene Level in Human Cells
Source: PLoS Genet. 2016 Feb 29;12(2):e1005901. doi: 10.1371/journal.pgen.1005901 (PMC4771717; doi:10.1371/journal.pgen.1005901)
Supplement: S8 Fig — The Rs, Rp and their P-values are shown on the top of each panel. (PDF) [file pgen.1005901.s013.pdf]

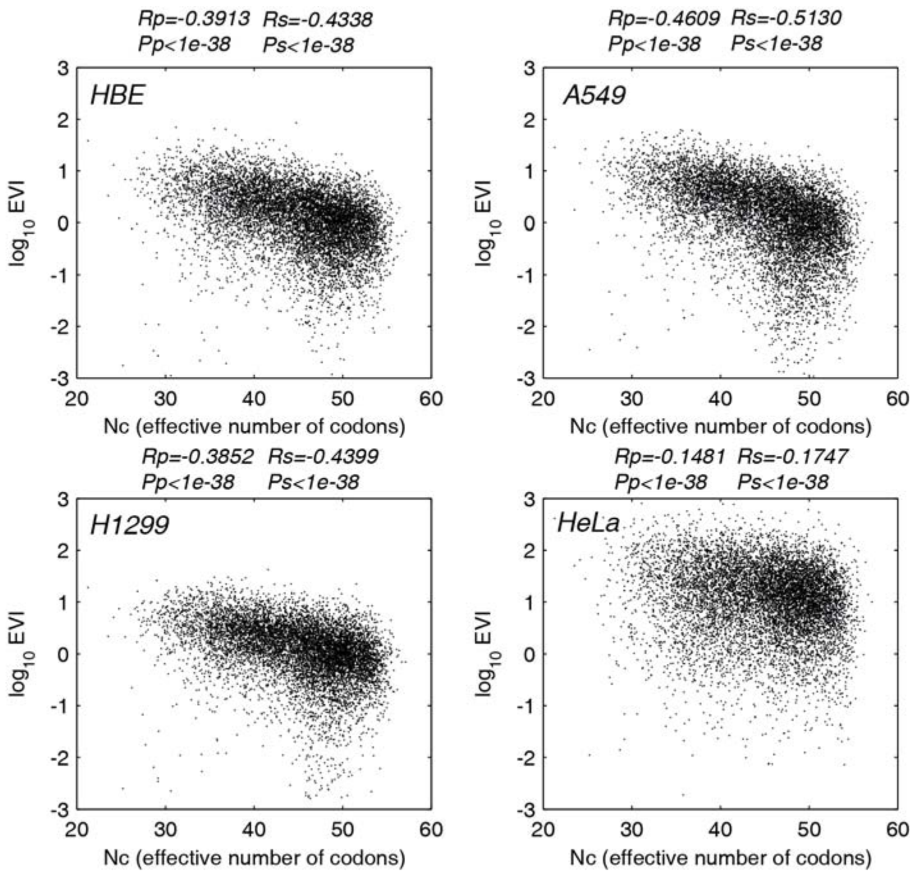

Figure S8: Correlation between EVI and Effective Number of Codons (Nc). Nc values for the genes were calculated according to [1, 2]. The *Rs*, *Rp* and their *P*-values are shown on the top of each panel.

1. Wright, F., *The 'effective number of codons' used in a gene.* Gene, 1990. **87**(1): p. 23-9.
2. Fuglsang, A., *The 'effective number of codons' revisited.* Biochem Biophys Res Commun, 2004. **317**(3): p. 957-64.
