# Supplementary material for: Genome-Wide and Experimental Resolution of Relative Translation Elongation Speed at Individual Gene Level in Human Cells
Source: PLoS Genet. 2016 Feb 29;12(2):e1005901. doi: 10.1371/journal.pgen.1005901 (PMC4771717; doi:10.1371/journal.pgen.1005901)
Supplement: S9 Fig — The sequence coverage range of each plot is indicated in the parentheses above each mRNA illustrations. The full length mRNAs are shown in thin lines, and the corresponding CDS regions are marked with thick lines. (PDF) [file pgen.1005901.s014.pdf]

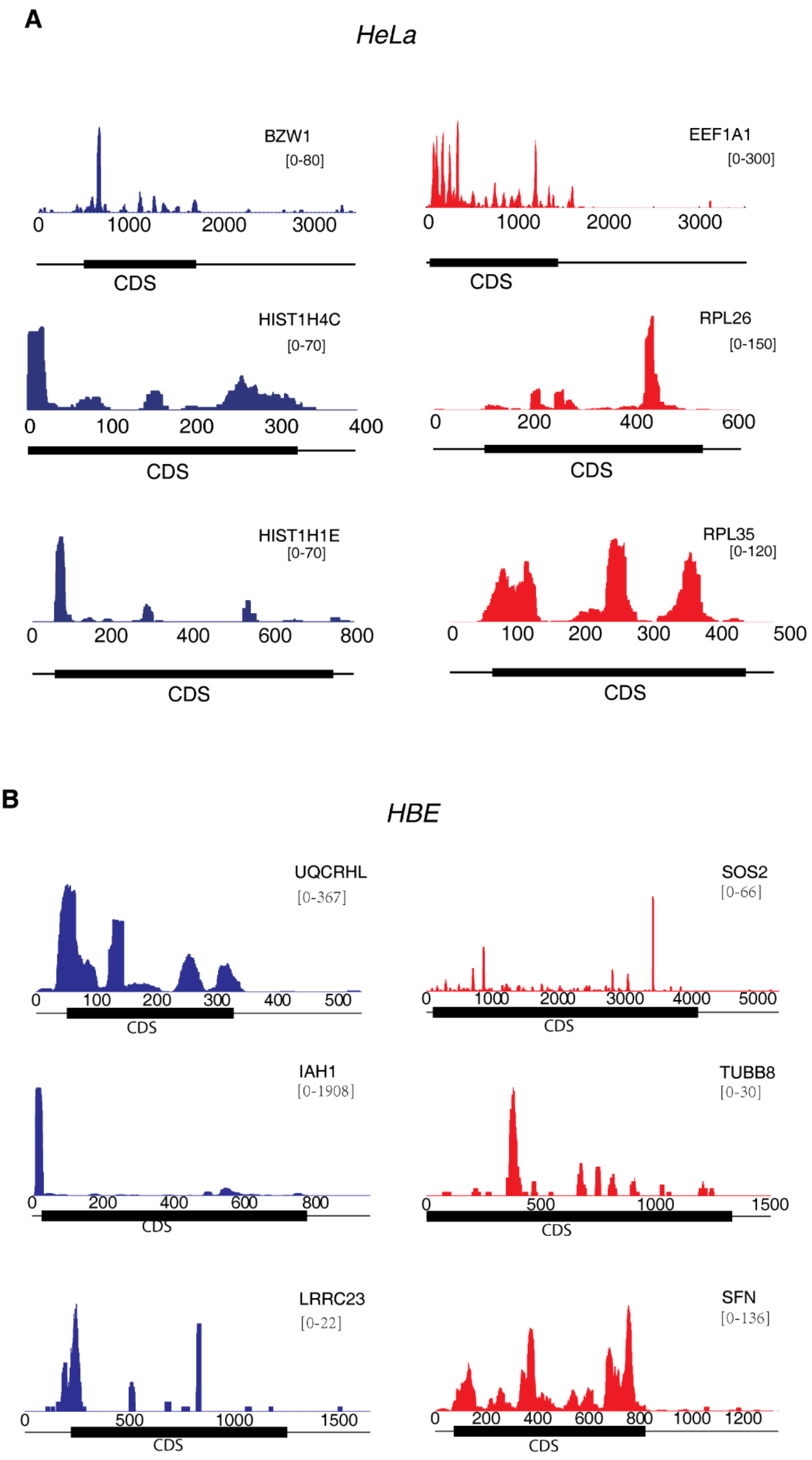
A
HeLa
BZW1
[0-80]
0 1000 2000 3000
CDS
EEF1A1
[0-300]
0 1000 2000 3000
CDS
HIST1H4C
[0-70]
0 100 200 300 400
CDS
RPL26
[0-150]
0 200 400 600
CDS
HIST1H1E
[0-70]
0 200 400 600 800
CDS
RPL35
[0-120]
0 100 200 300 400 500
CDS
B
HBE
UQCRHL
[0-367]
0 100 200 300 400 500
CDS
SOS2
[0-66]
0 1000 2000 3000 4000 5000
CDS
IAH1
[0-1908]
0 200 400 600 800
CDS
TUBB8
[0-30]
0 500 1000 1500
CDS
LRRC23
[0-22]
0 500 1000 1500
CDS
SFN
[0-136]
0 200 400 600 800 1000 1200
CDS

C

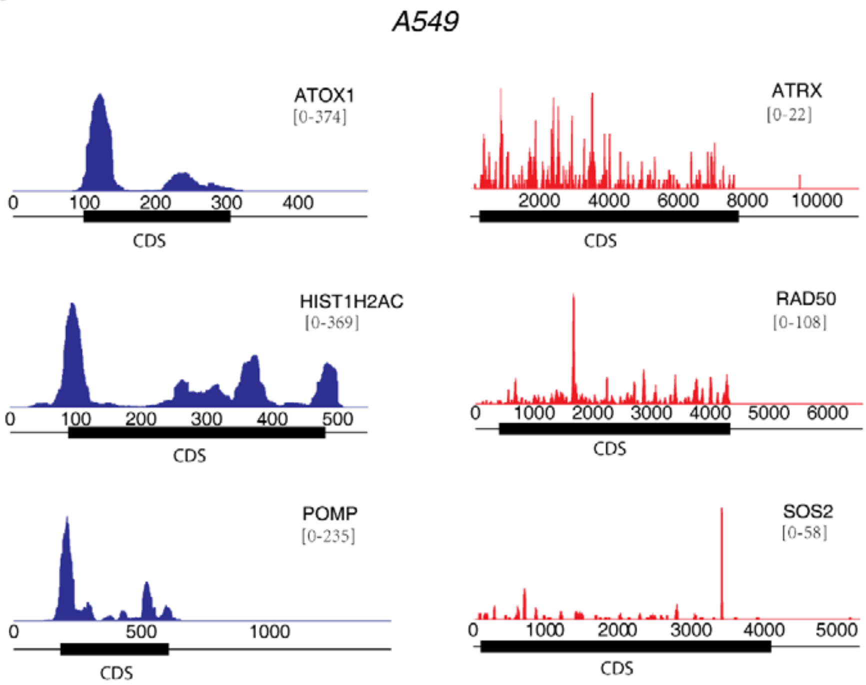

D

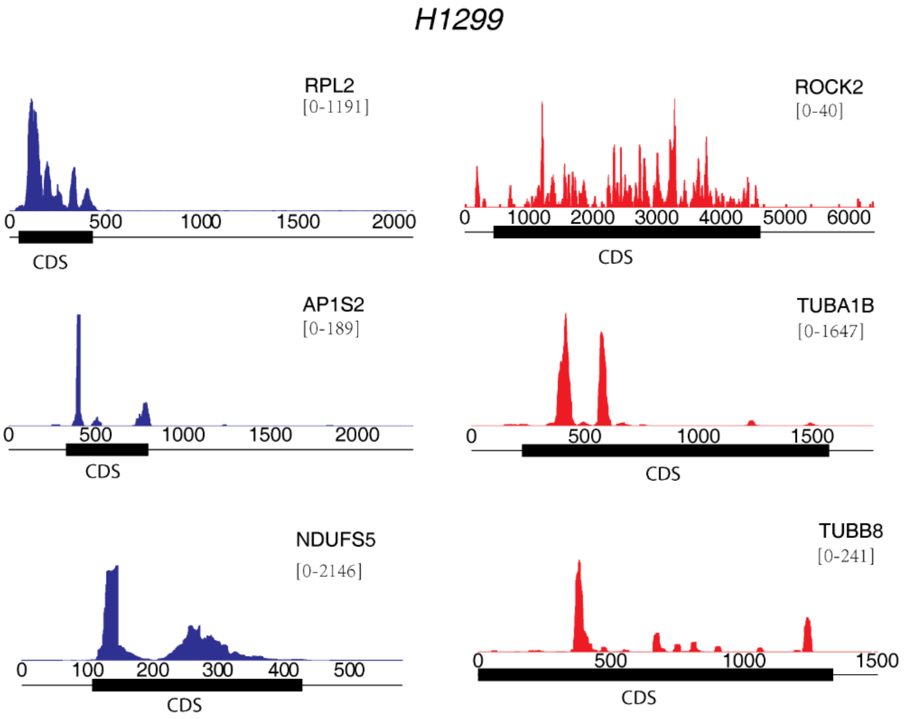

Figure S9. The RFP coverage of representative genes with high TR (blue) and low EVI (red) genes in HeLa (A), HBE (B), A549 (C) and H1299 (D) cells, respectively. The sequence coverage range of each plot is indicated in the parentheses above each mRNA illustrations. The full length mRNAs are shown in thin lines, and the corresponding CDS regions are marked with thick lines.
