# Supplementary material for: Genome-Wide and Experimental Resolution of Relative Translation Elongation Speed at Individual Gene Level in Human Cells
Source: PLoS Genet. 2016 Feb 29;12(2):e1005901. doi: 10.1371/journal.pgen.1005901 (PMC4771717; doi:10.1371/journal.pgen.1005901)
Supplement: S10 Fig — One-tailed KS-test were performed for each cell line under the null hypothesize that the low-EVI genes are not more stably folded than the high-TR genes. (PDF) [file pgen.1005901.s015.pdf]

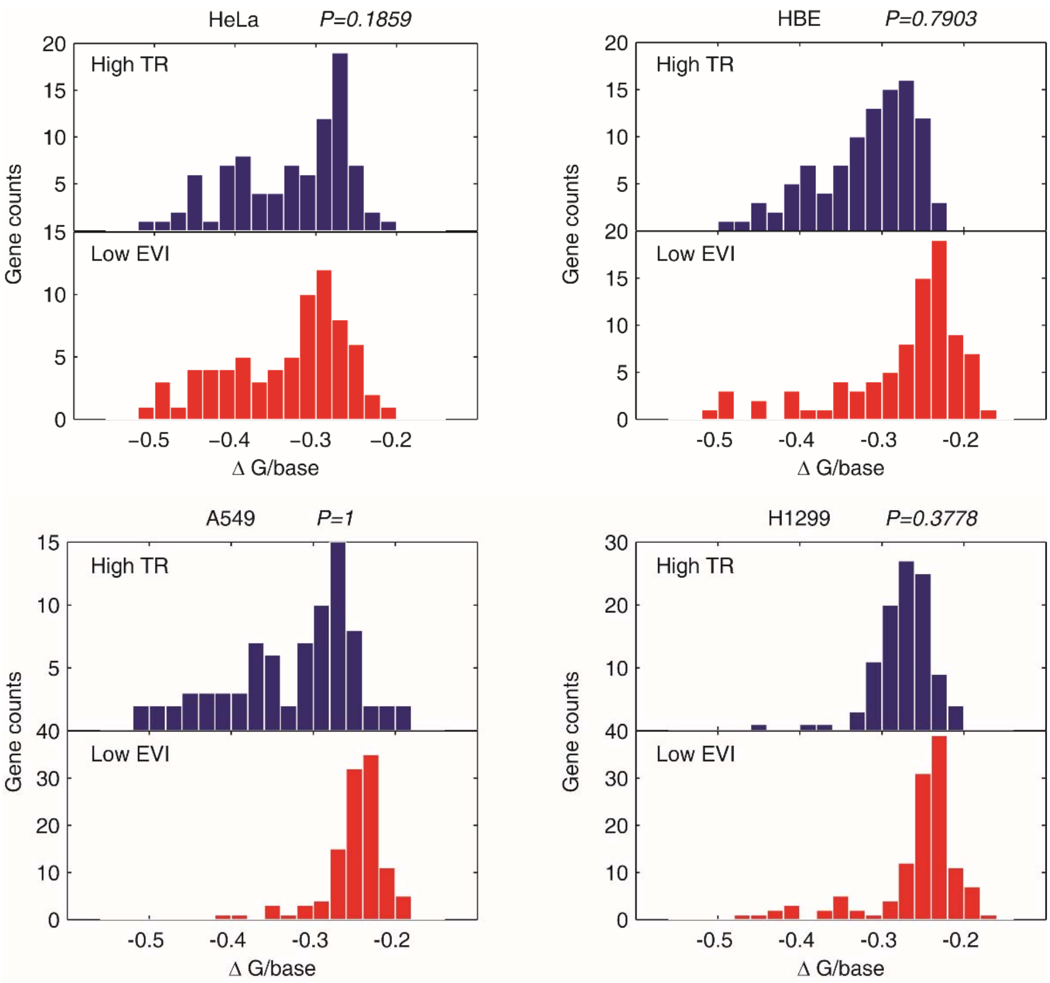

Figure. S10: The mRNA folding energy of the low-EVI and high-TR genes in four cell lines, calculated using MFOLD algorithm [1]. One-tailed KS-test were performed for each cell line under the null hypothesize that the low-EVI genes are not more stably folded than the high-TR genes.

1. Zuker, M., *Mfold web server for nucleic acid folding and hybridization prediction.* Nucleic Acids Res, 2003. **31**(13): p. 3406-15.
