# Supplementary figures and images for: Genome-Wide and Experimental Resolution of Relative Translation Elongation Speed at Individual Gene Level in Human Cells
Source: PLoS Genet. 2016 Feb 29;12(2):e1005901. doi: 10.1371/journal.pgen.1005901 (PMC4771717; doi:10.1371/journal.pgen.1005901)

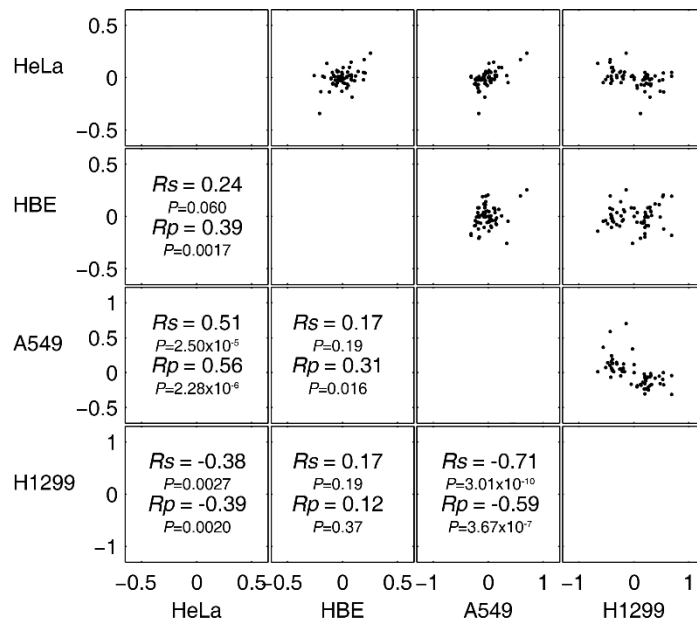

**Figure S11:** Plot matrix of the  $PS_{\text{High-TR}}$ . The  $R_s$ ,  $R_p$  and their  $P$ -values are indicated.

Supplement: S11 Fig — The Rs, Rp and their P-values are indicated. (PDF) [file pgen.1005901.s016.pdf]
