# Supplementary material for: Genome-Wide and Experimental Resolution of Relative Translation Elongation Speed at Individual Gene Level in Human Cells
Source: PLoS Genet. 2016 Feb 29;12(2):e1005901. doi: 10.1371/journal.pgen.1005901 (PMC4771717; doi:10.1371/journal.pgen.1005901)
Supplement: S12 Fig — The Rp, Rs and their P-values (Pp and Ps) are listed in the tables. (PDF) [file pgen.1005901.s017.pdf]

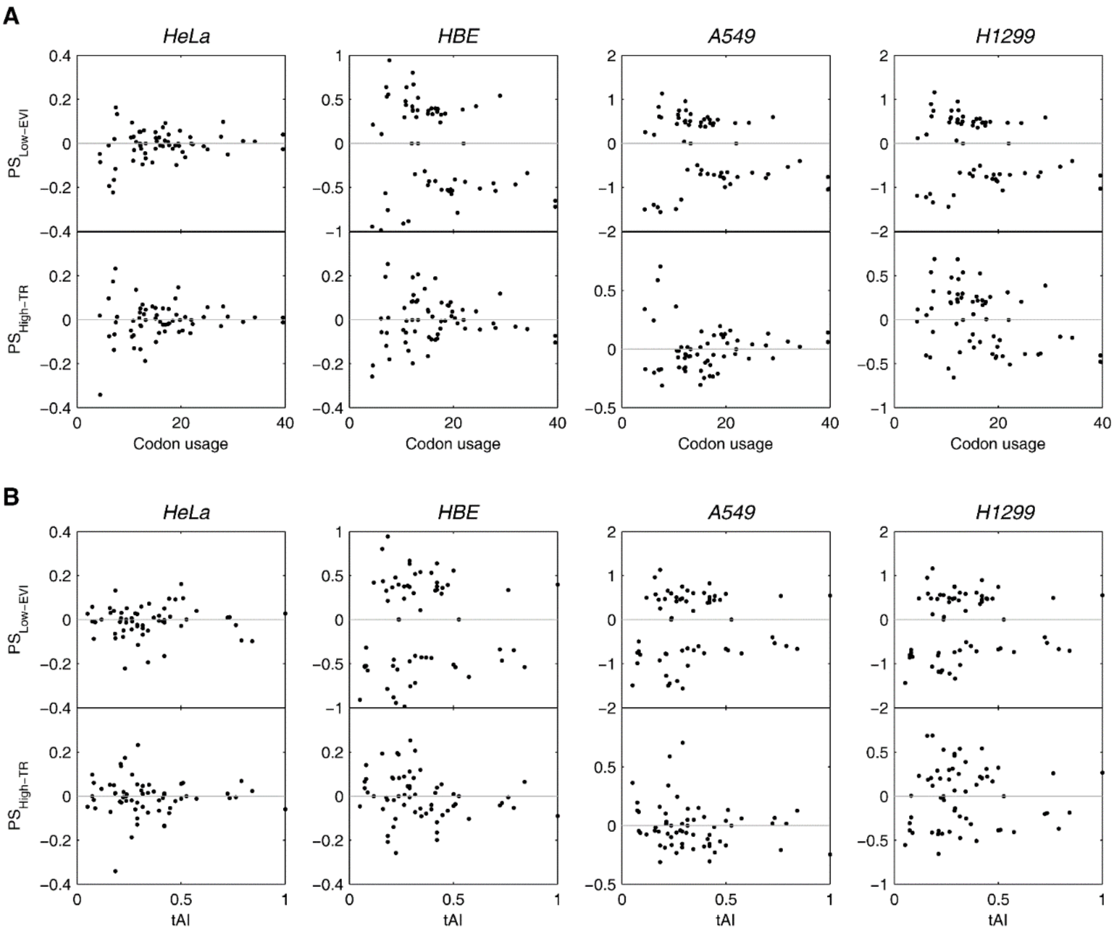

	$PS_{Low\text{-}EVI}$ vs. codon usage				$PS_{High\text{-}TR}$ vs. codon usage			
	HeLa	HBE	A549	H1299	HeLa	HBE	A549	H1299
Rp	0.194028	-0.28452	-0.21181	-0.2488	0.111336	0.013396	0.050073	-0.35292
Pp	0.134046	0.026258	0.101268	0.053169	0.392976	0.918385	0.701545	0.005275
Rs	0.142838	-0.23748	-0.22881	-0.23653	0.112242	0.086142	0.233172	-0.29489
Ps	0.272132	0.065346	0.076114	0.066465	0.389111	0.50919	0.070533	0.021049

	$PS_{Low\text{-}EVI}$ vs. tAI				$PS_{High\text{-}TR}$ vs. tAI			
	HeLa	HBE	A549	H1299	HeLa	HBE	A549	H1299
Rp	0.019665	0.068947	0.11584	0.116816	-0.03494	-0.19912	-0.18614	0.030701
Pp	0.88043	0.597509	0.373993	0.369952	0.789194	0.123937	0.150916	0.814305
Rs	0.038203	0.120666	0.162109	0.189932	-0.08628	-0.24404	-0.16343	0.107363
Ps	0.770049	0.354277	0.21196	0.142619	0.508487	0.058043	0.208206	0.410185

Figure S12. The $PS_{Low\text{-}EVI}$ and $PS_{High\text{-}TR}$ versus codon usage (A) and tAI (B) in HeLa, HBE, A549 and H1299 cells, respectively. The *Rp*, *Rs* and their *P*-values (*Pp* and *Ps*) are listed in the tables.
