# Supplementary material for: Genome-Wide and Experimental Resolution of Relative Translation Elongation Speed at Individual Gene Level in Human Cells
Source: PLoS Genet. 2016 Feb 29;12(2):e1005901. doi: 10.1371/journal.pgen.1005901 (PMC4771717; doi:10.1371/journal.pgen.1005901)
Supplement: S13 Fig — (PDF) [file pgen.1005901.s018.pdf]

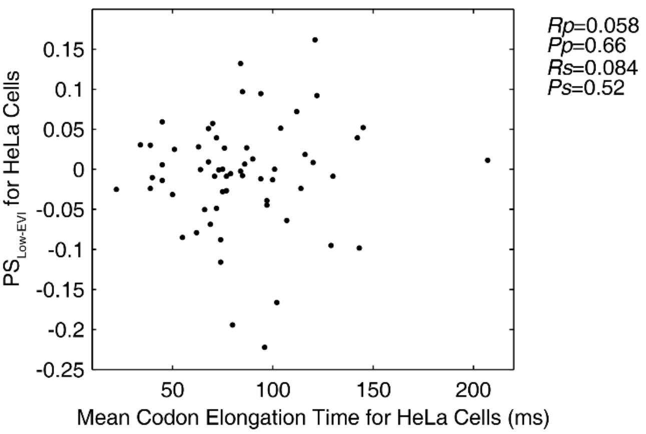

Figure S13: Correlation between $PS_{Low\text{-}EVI}$ (calculated in this study) and mean codon elongation time for HeLa cells (calculated in [1]).

1. Siwiak, M. and P. Zielenkiewicz, *Transimulation - protein biosynthesis web service.* PLoS One, 2013. **8**(9): p. e73943.
