# Supplementary material for: Genome-Wide and Experimental Resolution of Relative Translation Elongation Speed at Individual Gene Level in Human Cells
Source: PLoS Genet. 2016 Feb 29;12(2):e1005901. doi: 10.1371/journal.pgen.1005901 (PMC4771717; doi:10.1371/journal.pgen.1005901)
Supplement: S14 Fig — The Spearman R (Rs) and the P-values (Ps) were indicated. (PDF) [file pgen.1005901.s019.pdf]

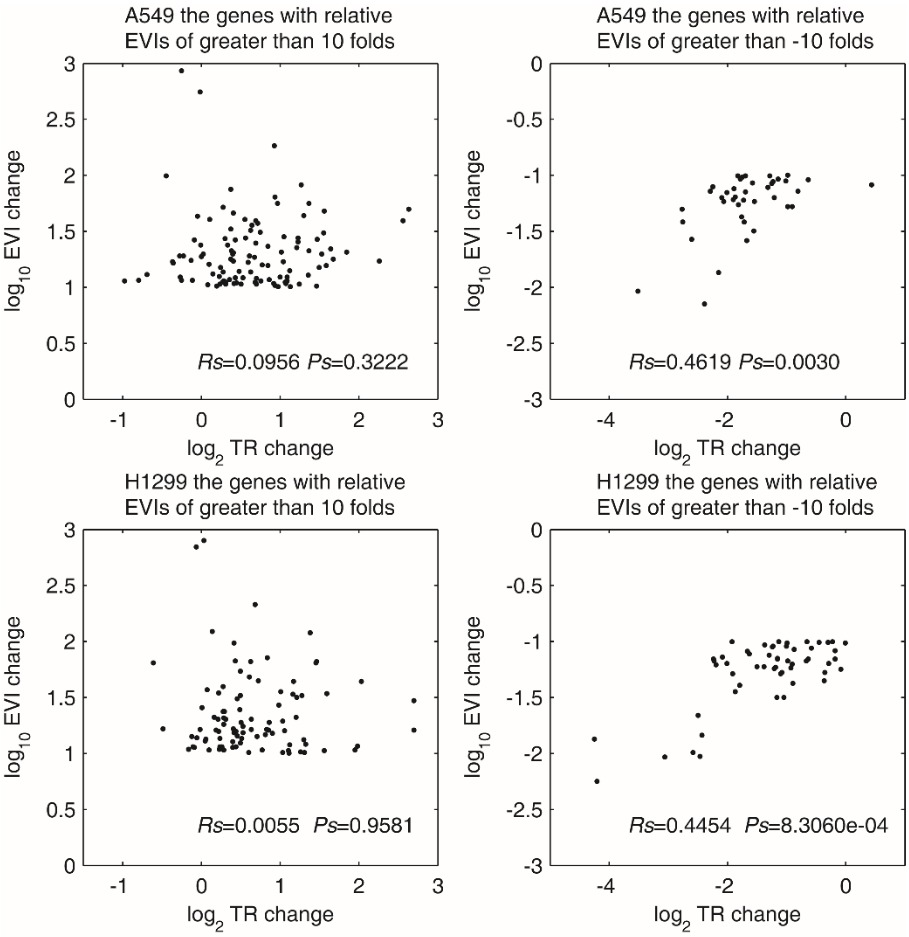

Figure S14: The EVI-TR correlation of the EVI up- and down-regulated genes in A549 and H1299 cells, respectively. The Spearman *R* (*Rs*) and the *P*-values (*Ps*) were indicated.
