# Supplementary material for: Genome-Wide and Experimental Resolution of Relative Translation Elongation Speed at Individual Gene Level in Human Cells
Source: PLoS Genet. 2016 Feb 29;12(2):e1005901. doi: 10.1371/journal.pgen.1005901 (PMC4771717; doi:10.1371/journal.pgen.1005901)
Supplement: S15 Fig — (PDF) [file pgen.1005901.s020.pdf]

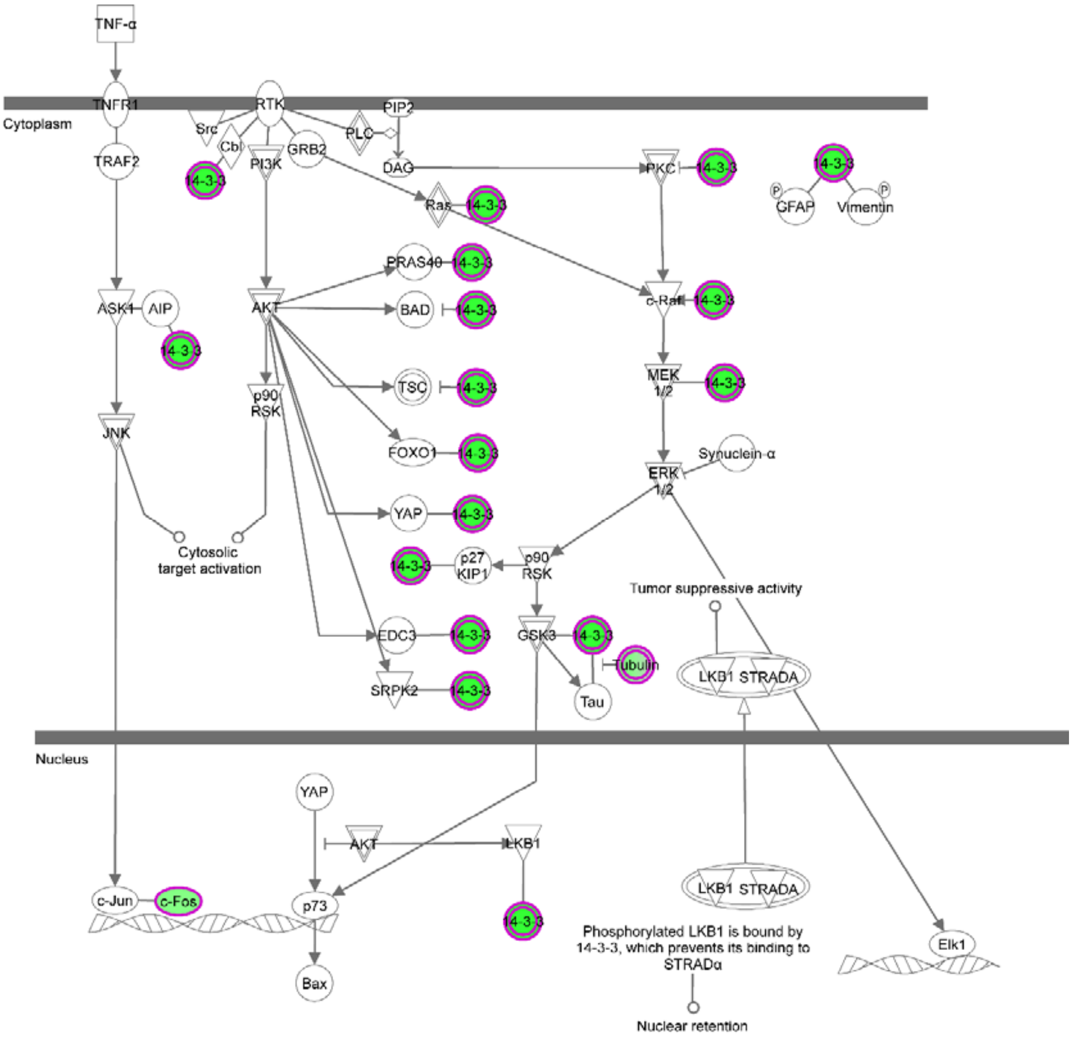

Figure S15. The top canonical pathway, 14-3-3 mediated signaling pathway, of the slow-translating genes, comparing lung cancer cells H1299 against the normal lung cells HBE.
