# Supplementary material for: Genome-Wide and Experimental Resolution of Relative Translation Elongation Speed at Individual Gene Level in Human Cells
Source: PLoS Genet. 2016 Feb 29;12(2):e1005901. doi: 10.1371/journal.pgen.1005901 (PMC4771717; doi:10.1371/journal.pgen.1005901)
Supplement: S16 Fig — (A) Plot matrix of mutual tRNA correlation of 7 human tissues normalized by the brain tRNA. (B) Rp and the –log10 p-values of the plot matrix. (C) The Rs and the #x2013;log10 p-values of the plot matrix. For (B,C), the numbers on the axes represent the tissues: 1 = liver, 2 = vulva, 3 = testis, 4 = ovary, 5 = thymus, 6 = lymph node, 7 = spleen. (PDF) [file pgen.1005901.s021.pdf]

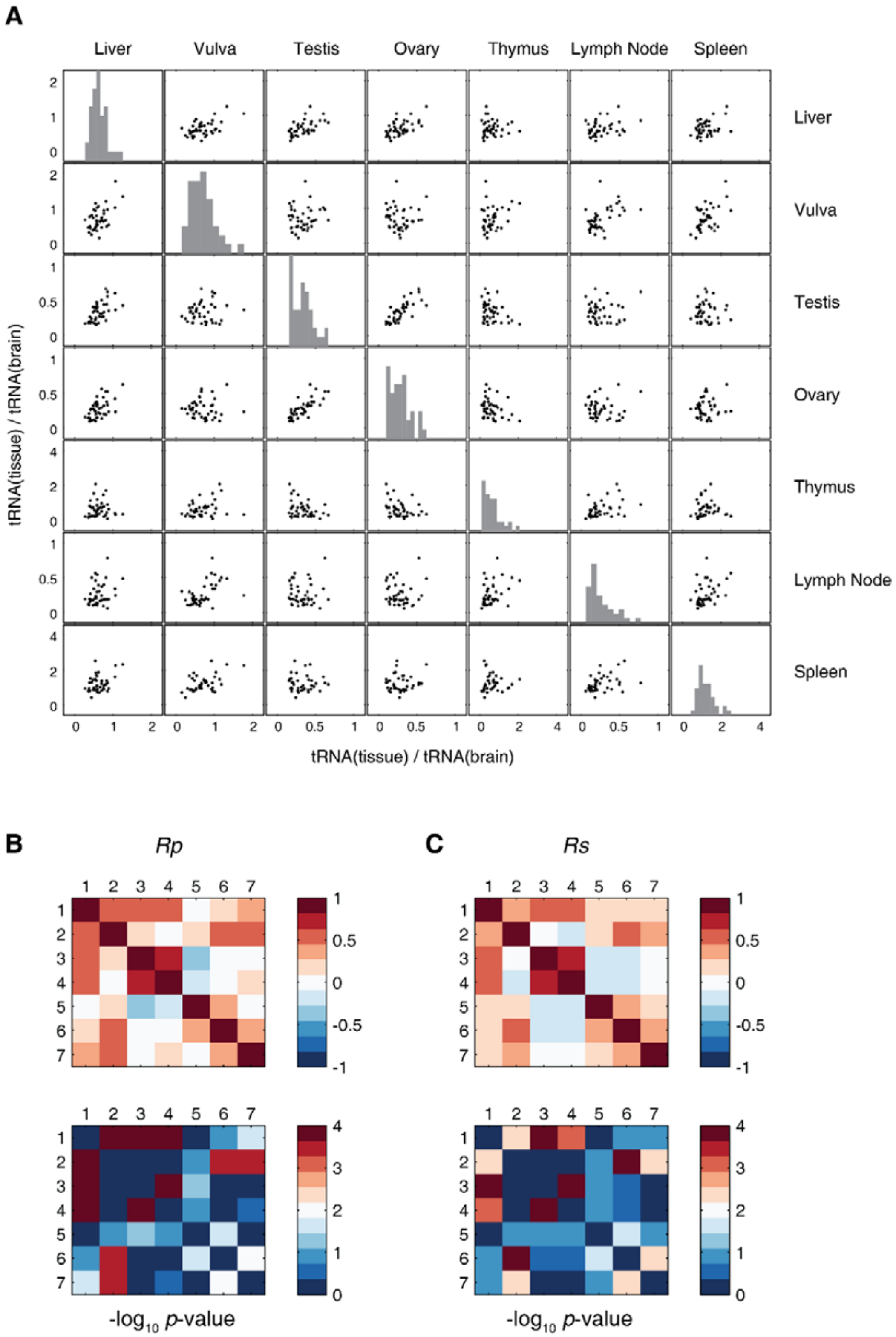

Figure S16: Mutual correlation of tRNA content of different human tissues. Data were obtained from [1]. (A) Plot matrix of mutual tRNA correlation of 7 human tissues normalized by the brain tRNA. (B) *Rp* and the –log10 p-values of the plot matrix. (C) The *Rs* and the –log10 p-values of the plot matrix. For (B,C), the numbers on the axes represent the tissues: 1 = liver, 2 = vulva, 3 = testis, 4 = ovary, 5 = thymus, 6 = lymph node, 7 = spleen.

1. Dittmar, K.A., J.M. Goodenbour, and T. Pan, *Tissue-specific differences in human transfer RNA expression.* PLoS Genet, 2006. **2**(12): p. e221.
