# Supplementary material for: Genome-Wide and Experimental Resolution of Relative Translation Elongation Speed at Individual Gene Level in Human Cells
Source: PLoS Genet. 2016 Feb 29;12(2):e1005901. doi: 10.1371/journal.pgen.1005901 (PMC4771717; doi:10.1371/journal.pgen.1005901)
Supplement: S17 Fig — Average ribosome read density profiles of all well-expressed genes with at least 200 RFP reads are shown plotted. (PDF) [file pgen.1005901.s022.pdf]

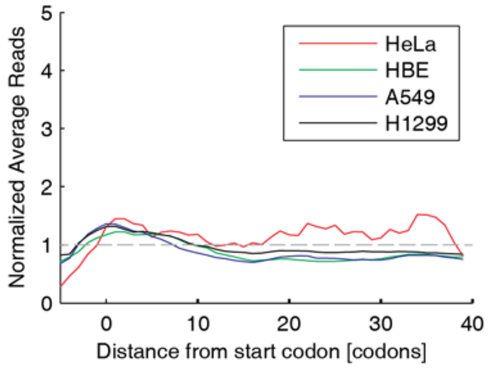

Figure S17: Metagene analysis of translation initiation of the 4 tested cell lines. Average ribosome read density profiles of all well-expressed genes with at least 200 RFP reads are shown plotted. The calculation was performed according to [1].

1. Ingolia, N.T., L.F. Lareau, and J.S. Weissman, *Ribosome profiling of mouse embryonic stem cells reveals the complexity and dynamics of mammalian proteomes.* Cell, 2011. **147**(4): p. 789-802.
